# ALDELE: All-Purpose Deep Learning Toolkits for Predicting the Biocatalytic Activities of Enzymes

**DOI:** 10.1021/acs.jcim.4c00058

**Published:** 2024-04-04

**Authors:** Xiangwen Wang, Derek Quinn, Thomas S. Moody, Meilan Huang

**Affiliations:** † School of Chemistry and Chemical Engineering, Queen’s University Belfast, Belfast BT9 5AG, Northern Ireland, U.K.; ‡ Department of Biocatalysis and Isotope Chemistry, Almac Sciences, Craigavon BT63 5QD, Northern Ireland, U.K.; § Arran Chemical Company Limited, Unit 1 Monksland Industrial Estate, Athlone, Co., Roscommon N37 DN24, Ireland

## Abstract

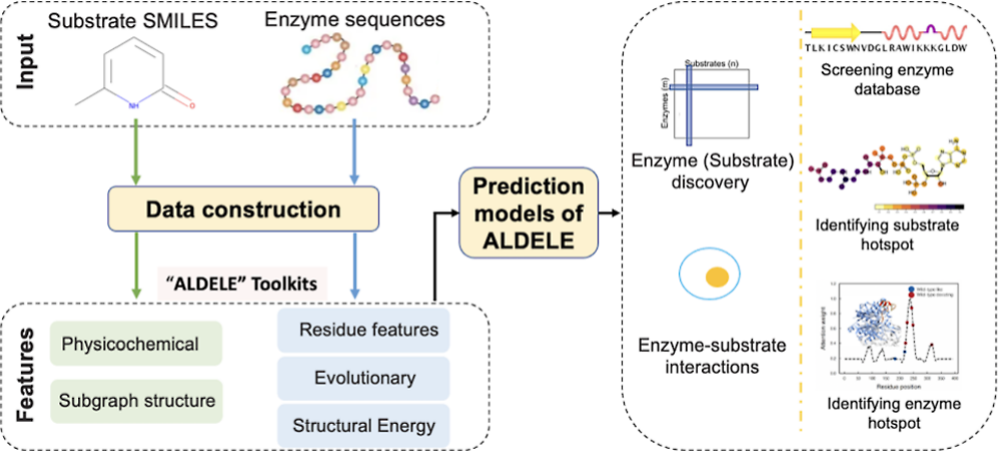

Rapidly predicting enzyme properties for catalyzing specific substrates is essential for identifying potential enzymes for industrial transformations. The demand for sustainable production of valuable industry chemicals utilizing biological resources raised a pressing need to speed up biocatalyst screening using machine learning techniques. In this research, we developed an all-purpose deep-learning-based multiple-toolkit (ALDELE) workflow for screening enzyme catalysts. ALDELE incorporates both structural and sequence representations of proteins, alongside representations of ligands by subgraphs and overall physicochemical properties. Comprehensive evaluation demonstrated that ALDELE can predict the catalytic activities of enzymes, and particularly, it identifies residue-based hotspots to guide enzyme engineering and generates substrate heat maps to explore the substrate scope for a given biocatalyst. Moreover, our models notably match empirical data, reinforcing the practicality and reliability of our approach through the alignment with confirmed mutation sites. ALDELE offers a facile and comprehensive solution by integrating different toolkits tailored for different purposes at affordable computational cost and therefore would be valuable to speed up the discovery of new functional enzymes for their exploitation by the industry.

## Introduction

Biocatalysis has gained significant attention in synthesizing diverse valuable fine chemicals due to its exceptional selectivity, mild reaction conditions, and eco-friendly nature.^1−3^ Nevertheless, numerous biocatalytic processes are hindered by low yields, restricted substrate scope, and insufficient stability of enzymes. By understanding the factors that influence biocatalytic efficiency, such as substrate specificity, stereoselectivity, and stability, researchers can develop new biotechnology to overcome these limitations.^4−6^ These rational approaches aim to expedite the screening process for optimal biocatalysts tailored to specific reaction steps within the biosynthetic pathway or the optimization of enzyme performance.

Traditional methods in screening potential enzymes for the desired biocatalytic reaction are mainly based on experiments, which are time-consuming, laborious, and limited by the experimental conditions. Computational virtual screening methods provide an time-efficient method for protein design and engineering.^7^ Large-scale screening can be conducted with simple homology searches^8^ by comparing enzyme sequences with those homologues used for precedent reactions.^9^ However, the proposed enzymes for a hypothetical reaction step often suffer from low catalytic efficiency, poor expressibility, or insufficient stability.^3^ Molecular docking^10,11^ and molecular dynamics simulations^12−15^ have been popularly used in determining the binding of various compounds with a given target protein to predict compound protein interactions (CPIs).

In this study, our goal was to develop a general machine learning model capable of predicting catalytic activity across all proteins. This tool is designed to help focus experimental efforts on pairs likely to exhibit a high catalytic efficiency. The model also can identify substrate scopes of identical enzyme or prediction enzyme performance on specific substrates. The data sets varied in size, encompassing scenarios with a limited range of substrate types and a diverse array of proteins, as well as situations with numerous substrate variations but a smaller set of candidate proteins. To achieve this, we are facing two primary challenges. First, numerical representations of each enzyme that maximally informs the downstream prediction task had to be established. To ensure broad applicability, these representations were based solely on the enzymes’ primary sequence, eliminating the need for additional features, such as protein structures and binding site characteristics. This involved capturing functional patterns related to the properties of interest. The generated features of substrates and proteins needed to strike a balance between not being overly simplistic or too sparse, particularly when dealing with smaller data sets. This leads to the second challenge: the data sets for biocatalysts vary in size, and the sizes of the experimental data sets with the functions of interest are usually small. Besides the prediction of the substrate scope for a single enzyme, there is also a need to tackle the catalytic effects of multiple proteins on a particular substrate.

The applications of machine learning to expedite CPIs are predominantly centered around drug discovery rather than biocatalyst screening. This focus has generated numerous reviews and substantial research.^16−18^ Machine learning has been extensively utilized to predict drug-target interactions (DTIs), focusing on both prediction accuracy and interpretability.^19,20^ Besides traditional machine learning techniques, deep learning techniques have recently been applied in DTI prediction recently. Yang et al.^21^ introduced MGraphDTA with a deep graph neural network (GNN) architecture composed of 27 layers for representing both local and global structures of drugs and a multiscale convolutional NNs (CNNs) for protein encoding. Wang et al. developed DeepDDS,^22^ a deep learning model that is trained by a large-scale training data set and an independent test set from AstraZeneca for predicting synergistic drug combinations for cancer treatment, which leverages GNNs and attention mechanisms to process the chemical structures of drugs and gene expression profiles. In contrast to previous methods that primarily used 3D structures of proteins or 2D features of compounds for prediction, sequence-based models do not rely on the structures and can potentially uncover new relationships and patterns in biological data. A sequence-based method DeepDTA^23^ employs CNNs to learn from 1D representations of target proteins and drugs. Another CNN method implemented transformers to capture residue-contact interaction information between sequential motifs.^24^ CON-Plex^25^ is a second-generation protein language model-based DTI model that leverages self-supervised contrastive learning to enhance discrimination between true drugs and similar decoys (drug-like molecules that do not bind to the protein). However, it is worth noting that all of the above machine learning models were built based on extensive and diverse drug-target databases. These databases usually contain a large number of protein–ligand interaction pairs, which provide a rich source of data for training and validating predictive models. In contrast, the field of biocatalysis often deals with more specific data sets tailored to particular reactions. The protein enzyme databases relevant to biocatalysis tend to be smaller and more focused. This specificity stems from the goal of finding highly efficient enzymes for specific chemical reactions, which is a key aspect of biocatalysis.

Recently, machine learning techniques have been used in biocatalyst screening, with the growing need to leverage biocatalysis for sustainable and efficient industrial processes that leverage biocatalysis. Traditional machine learning models, in the context of enzyme discovery, rely on the features obtained from databases for proteins^26^ and compounds,^27^ and the implicit residue-contact interaction information between them^28^ to predict binding affinity. Deep learning-based methods can be used to exhaustively exploit the local features of the inputs, allowing for the identification of the pattern of both compounds and proteins to predict their interactions. Jiménez et al.^29^ used a 3D CNN module with molecular representation of 3D voxels assigned to various physicochemical property channels. Besides these 3D CNN methods, Li et al.^30^ developed a method that is focused on the mutation site of variants and compared it with the wild-type structure to study the protein thermodynamic stability. These deep learning methods often improve scoring thanks to modeling long-range and multibody atomic interactions. Nevertheless, these methods rely on the actual 3D structures of CPI and are infeasible to apply for the large-scale predicted sequences. Sequence-based methods overcome the limited availability of structural data and mainly classify CPIs into binary classification (binding or not). Tsubaki et al.^31^ attempt to capture local CPI sites through neural attentions. BACPI^32^ utilized a bidirectional attention NN model to integrate the representations of proteins and compounds. Karimi et al. proposed^33^ DeepAffinity, which contains CNN and recurrent NN (RNN) modules for enzyme and substrates representations to predict quantitative CPIs. MONN^34^ utilized a pairwise module to learn both noncovalent interactions and binding affinities between compounds and proteins. Kroll et al.^35^ reported a substrate prediction model—“ESP” method with a transformer model trained on data using the augmentation method to determine whether a small molecule is a substrate for a given sequence; however, ESP cannot distinguish the catalytic efficiency, e.g., the conversion level of the enzyme for the positive substrates.

All of these prior models have some limitations in their applicability, either because they cannot be effectively applied to enzymes within individual functions or due to their dependence on large training data sets with a broad substrate scope for a single enzyme or enzyme family. It is evident that enzymes, even when sharing the same domain architecture or classified under the same Enzyme Commission number, can display considerable variability in catalytic efficiency toward the same substrates. This diversity in enzyme functionality underscores the need for a versatile and robust modeling approach, which has been unmet by prior models focusing on narrow enzyme scopes or large data sets constrained by enzyme family specificity. These approaches use simplified molecular-input line-entry system (SMILES) representations^36^ or fingerprints created with GNNs^37,38^ for numerical descriptions of the substrate molecule. The advantage of GNN-subgraph^31^ lies in its ability to capture more nuanced and localized information within molecular structures. While GNN-fingerprint generates a fixed-length overall representation for a molecule, the GNN-subgraph focuses on specific substructures within the molecule, allowing the model to capture local variations and complexities in the molecular structure that may be crucial for understanding intricate interactions or properties. In response to these challenges, we aimed to establish a cutting-edge model by integrating a tailored combination of GNN, CNN, and ANN, each serving a specific purpose in the representation and analysis of enzymes and substrates.

In this work, we proposed a novel deep learning model, ALDELE, designed for screening of biocatalysts. Specifically, compounds are represented by atom adjacency graphs and overall physicochemical properties, and proteins are represented by both global sequence information [including the features derived from amino acid (AA) sequences and position specific evolutionary information] and structure information derived from a weighted residue-centric scoring function. Enzymes and substrates are represented by features generated by deep-learning-based multitoolkits and a two-phase attention NN architecture. Then, the outputs of all toolkits are flattened, combined, and processed by a pairwise function^34^ to obtain represented descriptors for given protein-compound pairs. The combined descriptors were fed into a multilayer perceptron (MLP) for regression processing. This method enables the identification of local effective sites of compound atoms and protein AAs by highlighting the important regions in the structures. With this architecture, the ALDELE model can be used to predict CPIs. Particularly, ALDELE can be applied for fixed one-dimensional tasks; either the protein or the ligand is held fixed as the input, while the other component is subjected to variation. The fixed one-dimension tasks allow us to identify new enzyme sequences that can catalyze specific substrates or to identify compounds that can be catalyzed by specific enzymes. ALDELE offers a practical tool for screening newly designed sequences that are directly in line with industrial needs. Our research provides an automatic workflow for producing and selecting the optimal input features for constructing predictive machine learning models to be used for different tasks in biocatalysis.

## Methods

### Construction of the Benchmark Data Sets

ALDELE was evaluated with a wide range of benchmark biocatalyst data sets comprising 150 to 23,000 compound-protein pairs with regression or multiclass classification algorithm (summarized in Table 1), including the conversion by candida antarctica lipase B (CALB) mutants in the hydrolytic kinetic resolution,^39,39,40,40−44^ the collective *k*_cat_ data set from BRENDA and SABIO-RK databases,^45,46^ and the activities of thiolase,^47^ halogenase,^48^ glycosyltransferase,^49^ phosphatase enzymes,^50^ and the BVMO data set.^51−55^ These data sets offer valuable insights into enzyme-catalyzed reactions and have diverse applications. The CALB data set^39,39,40,40−44^ contains experimental data on substrate conversions and mutations of CALB, representing typical data in biocatalytic applications and enzyme engineering. Regarding the *k*_cat_ data set, the ultimate choice of 16,808 data points from the overall interaction count in BRENDA and SABIO-RK was adapted from the screening method described in the DLKcat paper.^56^ The data set was acquired, initially containing *k*_cat_ values, and then subjected to a cleaning process involving multiple filters to eliminate entries lacking UniportID, substrate name, protein sequence, and substrate SMILES information. Data containing special characters that could not be recognized were also excluded (Supporting Information S1 and github code). The thiolase data set^47^ focuses on OleA enzymes, unraveling catalytic mechanisms within the thiolase superfamily. The halogenase data set^48^ explores protein activities against chlorination and bromination substrates, enabling the discovery of new biocatalytic reactions. The glycosyltransferase data set^49^ categorizes enzyme activity, facilitating the study of glycosylation reactions. The phosphatase data set^50^ investigates enzyme–substrate interactions, while the BVMO data set^51−55^ sheds light on enzyme thermostability, essential for various oxidation reactions. These data sets collectively advance our understanding of enzyme-driven processes, impacting biocatalysis and industrial applications.

**Table 1:** Summary of Curated Data Sets with the Number of Unique Enzymes, Unique Substrates, Unique Pairs in Each Data Set, and the Pre-Processing Methods Used on Each Data Set

data set	Enz.	Sub.	CPI pairs	pre-processing
CALB conversion	96	25	306	percentage
collective *k*_cat_	7821	2669	16,808	Log2-transformation
thiolase activity	72	15	550	Log10-transformation
halogenase activity	42	62	2604	0.08 as the cutoff
glycosyltransferase activity	54	90	4298	multiclass classification
phosphatase activity^a^	22	108	2376	0.2 as the cutoff
BVMO thermostability^b^	152		152	

a The data set for substrate discovery task only.

b The data set for enzyme discovery task only.

Glycosyltransferase data were used in previous reports for machine learning evaluation, but they were simply binarized, i.e., each entry is labeled as either active (1) or inactive (0).^57^ Except for the *k*_cat_ data sets that is composed of 7821 proteins, the 3D structures of all other data sets were generated using RosettaCM.^58^ In our study, the data from benchmark data sets were preprocessed based on the values reported in the original experimental literature for regression or labeled into multiple classes for classification. (Details in Supporting Information S1).

### Model Architecture

**Figure 1:**
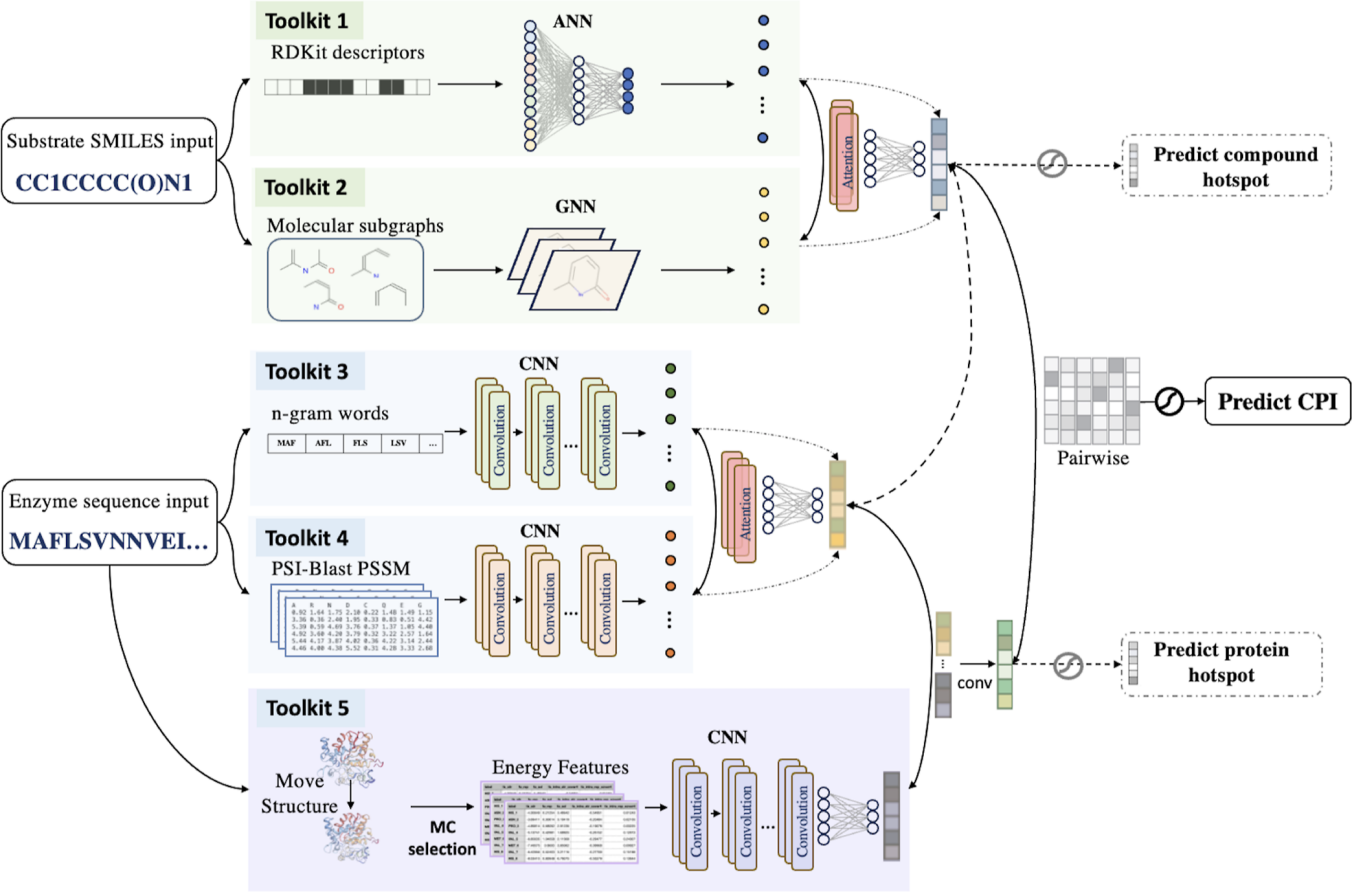
Overview of ALDELE architecture. The model architecture takes the protein sequences and substrate SMILES as the input with 5 toolkits to extract features. Toolkit 1: RDKit feature inputs of compounds for a NN module; Toolkit 2: SMILES inputs of compounds for a GNN module; Toolkit 3: “Words” inputs of protein sequences for a CNN module; Toolkit 4: PSSM inputs of protein sequences for a CNN module; Toolkit 5: protein structure-based features for a CNN module. The two-phase attention NN was applied to protein-based features (readout of toolkit 1 and 2) and ligand-based features (readout of toolkit 3 and 4) separately to extract two sets of vectors representing the given protein sequence or ligand. The biological interpretation is provided with the representing vectors. The dashed line represents the vectors can be directly taken from the previous layer if the other toolkit is not used in the model. A pairwise interaction module was applied for obtaining a combined vector of the interactions between the input protein and ligand, followed by a MLP for prediction.

The learning framework for the prediction of biocatalysts is illustrated in Figure 1. For each pairwise combination, our model comprises two main architectures containing 5 toolkits to extract features from protein and substrates, respectively. The Toolkits consist of: (1) an artificial NN module for extracting the features of RDKit descriptors of the whole compound, (2) a GNN module for extracting the features of compound from a given r-radius molecular graph, (3) a CNN module for extracting the AA features of protein sequences, (4) a CNN module for extracting the AA features of position-specific scoring matrix (PSSM) that represents the evolutionary history of a given protein sequence, and (5) a CNN module for extracting weighted energy features from protein structures. The two-phase attention NN was applied to protein sequence-based features and ligand-based features, respectively; the former was then combined with structure-based features to extract vectors representing the given proteins, and the latter was directly used as ligand representations. The representation learned can be semantically rich and improve multiple downside tasks. A pairwise interaction module was then constructed for the learned features of proteins and ligands to predict the interactions. Therefore, ALDELE considers protein sequence, structural, and evolutionary information to learn the representations of sequence variants, alongside the subgraphs and overall physicochemical properties of ligands.

The toolkits employed in ALDELE are selectively combined based on the data set and computational constraints, allowing for a tailored and efficient prediction process. Here, we developed models with various combinations to compare substrate representations, protein representations, and the presence of an attention module for data sets of different sizes and distributions. Six models with different combinations of modules were developed and are denoted as follows: CPI-Model 1 is based on Toolkits 2 and 3; CPI-Model 2 is based on Toolkits 2 and 4; CPI-Model 3 is based on Toolkits 2, 3, and 4; CPI-Model 4 is based on Toolkits 1, 2, and 3; CPI-Model 5 is based on Toolkits 1, 2, 3, and 4; and CPI-Model 6 is based on Toolkits 1, 2, 3, 4, and 5. In the fixed one-dimension enzyme discovery models, only the features of proteins were used as the input and in the fixed one-dimension substrate discovery models, only the features of substrates were used as the input. (The structures of above models were summarized in Supporting Information, S2.)

The toolkit combinations were built as follows: the difference of CPI-Model 1 and CPI-Model 4 reveals the impact of incorporating substrate global information on the model’s optimization Toolkit 2 utilizes GNN-subgraph, a validated method for describing substrate features, while Toolkit 1 leverages RDKit to generate overall physicochemical information, The performance evaluation of CPI-Model 1 and CPI-Model 4 provides insights into how the model optimization varies with the inclusion of substrate global information, contributing to a comprehensive understanding of the model’s effectiveness in capturing substrate characteristics. Comparing CPI-Models 1, 2, and 3 is essential to assess the impact of different protein description methods or combinations of two sequence-based description methods on model performance: Toolkit 3 employs a widely applied numerical encoding method based on deep learning representations of AA sequences, while Toolkit 4 generates PSSM through psi-blast, incurring substantial computational time, particularly for large databases. CPI-Model 5 combines two substrate and two protein representation toolkits, incorporating a two-phase attention model that can be compared with the one-phase attention models of CPI-Model 3 and CPI-Model 4. CPI-Model 6, an extension of CPI-Model 5, incorporates Toolkit 5, which relies on structure-based energy term descriptors produced by RosettaCM. Due to the lack of structural information on these data sets, generating structural features requires additional computation time. The evaluation across data sets of varying sizes and distributions aims to determine the effectiveness of these protein description strategies and whether the potential improvement in model performance justifies the computational overhead associated with generating PSSM and energy terms, especially in the context of large databases. The two-phase attention is applied to protein and compound representations separately in CPI-Model 5 and 6, and all of the ALDELE models contain a pairwise module for enzyme–substrate interactions. This two-phase attention model allows ALDELE to identify and weigh the importance of different regions in the protein and substrate, more precisely than 1D attention model.

#### Toolkit 1: NN Representation of Substrates based on Whole Compound Properties

RDKit, an open-source chemical informatics software package, version 2020.03^59^ was used to get a set of 200 molecular descriptors for each compound. These descriptors are conformation-independent and can be categorized as either (computed) experimental properties (e.g., molar refractivity, log *P*) or theoretical descriptors derived from a symbolic representation of the molecule (e.g., 1D compositional properties such as heavy atom counts, bond counts, and molecular weight or 2D topological properties such as fragment counts, topological polar surface area, and connectivity index). Any features with invariant values across the data set, or with null valued (i.e., infinite or not computable), were excluded. The full list of descriptors is included in Supporting Information, S3-1. These ligand-based features on all the data sets will then be passed to a feature selection based on their ranked importance. The top 10 RDKit descriptors were retained and converted as the input vectors of the NN with a nonlinear activation function ReLU. The output *y*_RDKit_ is a set of hidden vectors at time step t (denoted as *r*_*i*_^(*t*)^) for the input compound.

#### Toolkit 2: GNN Representation of Substrates based on Molecular Graphs

**Figure 2:**
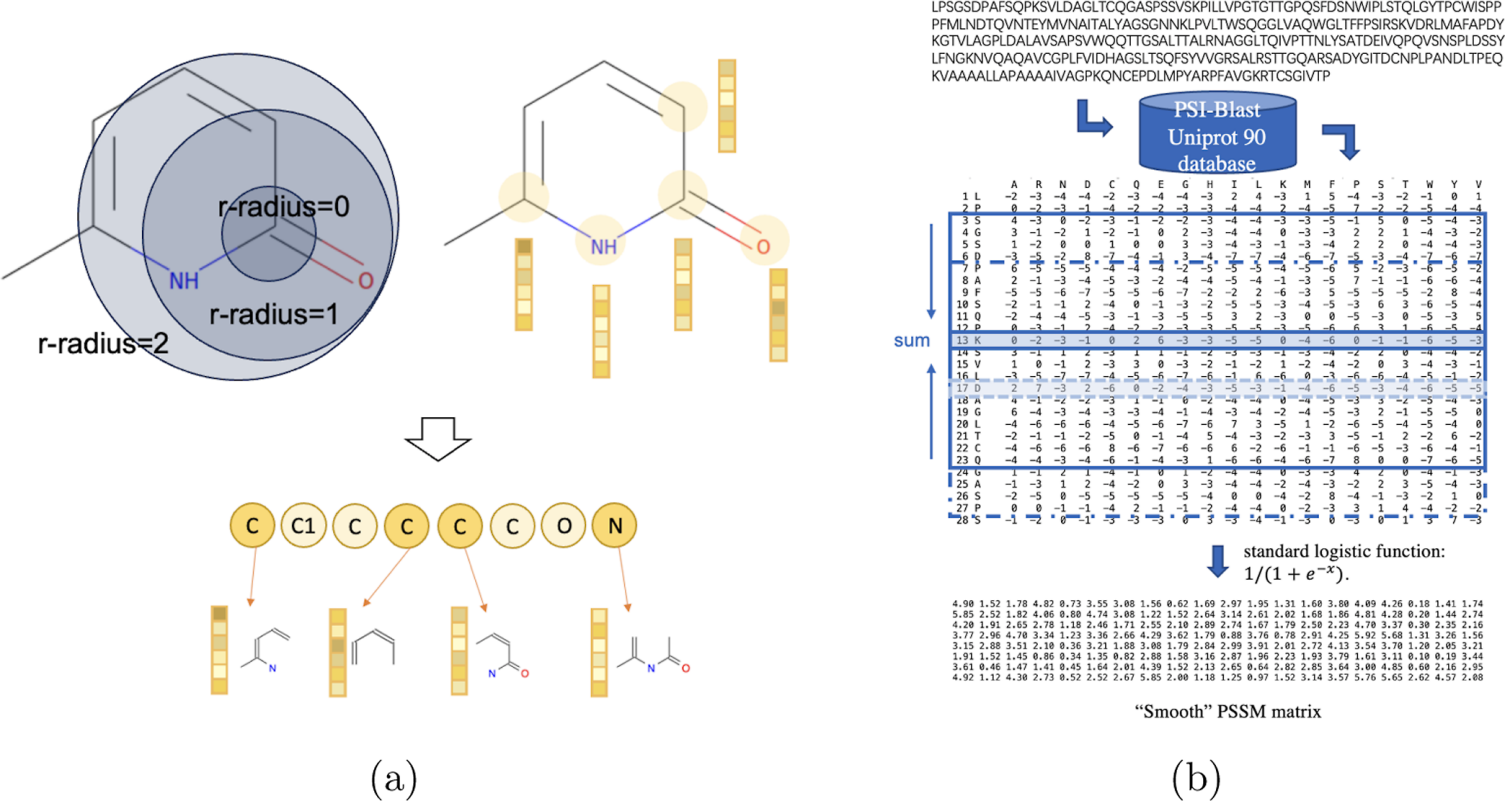
Ligand encoding method and protein encoding method. (a) Converting molecular structure into graph embedding by representing a molecule with different r-radius for graph features and readout of ligand. (c) Outline of the “smooth” method to generate the PSSM feature from a protein sequence input.

Substrates are represented by molecular graphs, where the vertices are atoms and the edges are chemical bonds. For each type of r-radius vertex and r-radius edge, embedding r-radius subgraphs were employed to get the vector representations, and all parameters are trained by backpropagation during supervised learning. Given the molecular graph of a substrate, the GNN learning uses the graph representation *G* = (*V*, ε) (where *V* is the set of vertices and ε is the set of edges of a compound) as the input and processes the data via a nonlinear function ReLU. In a molecule, *v*_*i*_ ∈ *V* is initially represented by the ith atom, which is the concatenation of one-hot encodings representing the atom type, and *e*_*ij*_ ∈ ε is the chemical bond between the concatenating atom and the surroundings, which represent the relations of the corresponding atom. The r-radius subgraph for vertex *v*_*i*_ was defined as *v*_*i*_^(*r*)^ = (*V*_*i*_^(*r*)^, ε_*i*_^(*r*)^). Two transitions were then included in the GNN, including vertex transitions and edge transitions, to ensure that the embeddings of vertices and edges are considered equally and updated simultaneously such that global information can be gradually gathered. The final output of the GNN is a set of real-valued molecular vector representations by the transition function, i.e., *V* = {*v*_1_^(*t*)^, *v*_2_^(*t*)^, ... , *v*_*V*_^(*t*)^} where |*V*| is the number of vertices in the molecular graph of substrates (Figure 2a).

#### Toolkit 3: CNN Representation of Proteins based on N-Gram Vectors

The protein sequences are split into overlapping n-gram AAs, which encode protein sequences in input vectors, so-called “words” of AAs. Because there are 20 types of AAs, the total number of possible n-grams is 20^*n*^. The value of n is set as 3 according to previous studies^31,34,56^ to keep the vocabulary size tractable and avoid low-frequency words in the learning representations. Given a protein sequence *S* = *x*_1_, *x*_2_, *x*_3_, ... , *x*_*S*_, where *x*_*i*_ is the d-dimensional embedding of the ith word and |*S*| is the sequence length, all words can be translated to randomly initialized embeddings, which is referred to as “word embeddings”. When receiving the “word” input features of the protein sequences, a CNN module is used to process the data to give low-dimensional real-valued vector. The CNN module uses a filter function to compute a hidden vector from the word embeddings (input features) and weight matrix (learning parameter) as follows
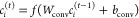
1where *f* is a nonlinear activation function, ReLU, *W*_conv_ is the weight matrix, and *b*_conv_ is the bias vector. Note that all filter functions are implemented by NNs. Before being fed into each convolutional layer, the input is zero-padded to ensure that the number of output features remains fixed. As a result, a set of hidden vectors *C* = {*c*_1_^(*t*)^, *c*_2_^(*t*)^, ... , *c*_|*C*|_^(*t*)^} are obtained.

#### Toolkit 4: CNN Representation of Proteins based on PSSM

Protein sequences are represented by PSSM^26,60,61^ that contains evolutionary information on the probability of mutating to 20 types of AA at each position of protein sequence and is obtained by comparing with a large database Uniprot90 using PSI-BLAST.^62,63^Figure 2b illustrates the PSSM generating approach. To consider the neighbors of each AA, an encoding scheme is used to incorporate the influence of the surrounding residues on the central AA sites. A sliding window of size *w* is used to get the “smoothed” PSSMs, where each row vector is represented by the summation of (*w* – 1)/2 upstream and downstream row vectors. The profile matrix elements are scaled to the required 0–1 range by using the standard logistic function: 1/(1 + *e*^–*x*^). Toolkit 4 was designed based on the PSSM profiles to incorporate the sequence conservation information. The final scaled PSSM of each sequence are updated through convolutional layers with a ReLU activation function to obtain a set of hidden vectors labeled *M* = {*m*_1_^(*t*)^, *m*_2_^(*t*)^, ... , *m*_|*M*|_^(*t*)^}, where |*t*| is the number of AA residues. The final output features *y*_PSSM_ were obtained from the set of hidden vectors as follows
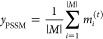
2

#### Toolkit 5: CNN Representation of Protein Structure-Based Features

When the structures of variant enzymes cannot be found in the Protein Data Bank,^64^ their structures can be obtained by computational methods like AlphaFold2^65^ and Rosetta.^66^ Toolkit 5 was designed based on the Rosetta energy terms to incorporate the structure information. Protein structure-based features are extracted from Rosetta simulations by comparing the structures of the wild-type and variant enzymes. Quantifying the effect of mutations on enzyme structures and their functions presents a challenge. The Rosetta Energy function combines physical and statistical potentials to approximate the energetic stability of protein structures and is decomposed into individual scoring terms.^67^ In the protocol, the native residues are mutated to generate the variants, the variant structures are refined, and the weighted Rosetta Energy score terms are calculated for the output structures. The full list of the features and descriptors is included in Supporting Information S3-2. The structure-based features were normalized by comparing scores to scores derived from Rosetta-relaxed ensembles of its wild-type protein.^68^ The 20 AAs were encoded as 1 to 20. For a given protein structure, each residue of the structure was represented as an AA type and a vector of energy features. A matrix with size (N*(*F* + 1)) was generated where N is the number of residues and F is the number of energy features. The comparisons between the variants in relation to the wild type were then used as the inputs for a CNN block. A deep learning model requires that input features of all samples have the same length for each block. Principal component analysis was utilized for dimensionality reduction, and then the input features with the same dimension were processed by other convolutional layers with a ReLU activation function to obtain a set of hidden vectors labeled *E* = {*e*_1_^(*t*)^, *e*_2_^(*t*)^, ... , *e*_|*E*|_^(*t*)^}, where |*t*| is the number of AA residues. The final output features *y*_energy_ were obtained from the set of hidden vectors as follows
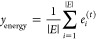
3

#### Explanation of Neural Network Choices for Toolkits

In the development of the ALDELE model, the choice to utilize specific types of NNs (GNN, CNN, and ANN) is strategic, aiming to leverage the unique strengths of each network in handling biological data and ensure that these tools align with the objectives of our study. The GNN for processing the subgraph representations of compounds is motivated by its capability to effectively encode the graph-like, non-Euclidean data of molecular structures, encompassing the connectivity and topological features of atoms. Such representations are crucial for understanding and predicting the interactions between compounds and proteins. Through graph embedding, GNNs capture complex local and global molecular patterns, which are predictive of which groups of molecules affect the interaction with proteins. CNNs are used in Toolkits 3 and 4 to extract features from protein sequences. The success of CNNs in matrix recognition tasks has inspired their application to sequence data. By utilizing n-gram representations of AAs, CNNs can capture local sequential patterns, which are essential for understanding the functions of proteins and their biochemical properties. Furthermore, CNNs are used to process PSSMs generated via PSI-Blast, providing a rich matrix representation of the evolutionary information on proteins. ANNs are utilized in Toolkit 1 to encode compounds based on their overall physicochemical properties, which learn 1D features of compound molecules from both theoretical and experimental properties,. This network structure is effective in capturing the overall physicochemical characteristics that influence compound activity, such as the molecular charge distribution, hydrophobicity, size, and shape. In summary, each NN framework within the ALDELE model is meticulously selected and optimized to work in synergy, enhancing the prediction accuracy of complex enzyme–substrate interactions. This integrated multinetwork approach not only enhances the model’s generalizability but also improves its accuracy and interpretability in predicting biocatalytic activities.

#### Prediction of Biocatalyst Properties of Enzymes

The significance of the subsequences in the protein that is important for the property was evaluated based on the *y*_PSSM_ evolutionary information using the weights *c*_*i*_^(*t*)^. Given a sequence vector *y*_PSSM_, a set of hidden vectors of subsequences in a protein is obtained *C* = {*c*_1_^(*t*)^, *c*_2_^(*t*)^, ... , *c*_|*C*|_^(*t*)^}. Such weights can be modeled using NNs, i.e., the neural attention mechanism,^69^ and were computed as dot product-based scalar values using the following equation as
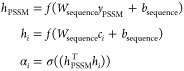
4where *f* is a nonlinear activation function, ReLU, *W*_sequence_ is the weight matrix, and *b*_sequence_ is the bias vector. The weight value α_*i*_ indicates vector importance of PSSM on the subsequence of a protein. Based on the attention weights, the weighted sum of *h*_*i*_ was obtained by the following equation
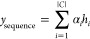
5

Introducing the attention mechanism allows us to evaluate the influence of the PSSM on the protein sequences rather than obtaining a simple summation. The final outputs are sequence-based protein representations. The attention module is also used on the RDKit descriptors hidden vectors *y*_RDKit_ and a set of subgraph hidden vectors *V* = {*v*_1_^(*t*)^, *v*_2_^(*t*)^, ... , *v*_*V*_^(*t*)^} to get the weight matrix *W*_compound_ for compounds. The attention weight can be regarded as a measure of importance of the feature at each position (e.g., an atom or a residue), and thus, such an attention mechanism enables the interpretation of the effect of each modification.

The sequence-based features from the attention modules and the structure-based features were then concatenated and transformed into a compatible space by two single-layer NNs to obtain the protein representations and obtain the updated weight matrix *W*_protein_. These protein representations and substrate representations were then used as the input to a pairwise module. The module is used to predict the pairwise interactions between an atom *v*_*i*_ of the compound and a residue *c*_*j*_ of the protein, normalized by a sigmoid function, as follows
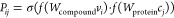
6where *f* is a nonlinear activation function, *W*_compound_ and *W*_protein_ are the weight matrix, and σ is the sigmoid function σ(*x*) = 1/(1 + *e*^–*x*^). Given a set of all compound-protein pairs and the labels in a training data set, the training objective is to minimize the loss function , given as the cross-entropy loss as follows
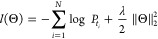
7where Θ is the set of all weight matrices and bias vectors in our pairwise interaction, GNN, CNN, and NN, the embeddings of r-radius vertices and edges, and the embeddings of n-gram AAs. *N* is the total number of molecule–protein pairs in the training data set, *t*_*i*_ is the ith label, and λ is an L2 regularization hyper-parameter. Then, Θ was trained by backpropagation.

The performance of the machine learning models was evaluated by the root mean squared error (*r*.*m*.*s*.*e*.) and *R*-squared (*R*^2^), calculated by scikit-learn v.0.23.2 (https://scikit-learn.org/stable/). The coefficient of determination (*R*^2^) is used to describe the quality of the hyperparameter on deep learning performance. *R*-squared measures the correlation between established model and the dependent variables. Finally, the optimal hyperparameters was used to train the deep learning model.
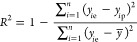
8where *y*_*i*p_ is the predicted value, *y*_*i*e_ is the experimental value, *y̅* is the average of the experimental values and *n* is the total number of items in the data set (validation data set or test data set).

During the training process, each data set was shuffled at the first step, and then the 5-fold cross-validation method was used. The minimal *r*.*m*.*s*.*e*. with the cross-validation for model checking is given as
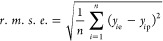
9

If the predicted responses are sufficiently close to the true values, *r*.*m*.*s*.*e*. would be small. On the contrary, if the predicted and true responses differ substantially, the *r*.*m*.*s*.*e*. would be large.

## Results and Discussion

### Model Performance for CPI Tasks on Biocatalytic Data Sets

To study the predictive ability of interactions between enzymes and substrates, we constructed ALDELE models for the regression task on Thiolase, Halogenase, collective *k*_cat_, and CALB conversion data sets. The effect of hyperparameters on deep learning performance was evaluated by learning curves (Supporting Information, S4). The optimal hyperparameters are as follows: r-radius: 2; n-gram: 3; PSSM sliding window size: 21; vector dimensionality: 10; number of layers in GNN: 3; number of layers in NN: 3, and number of layers in CNN: 3.

**Figure 3:**
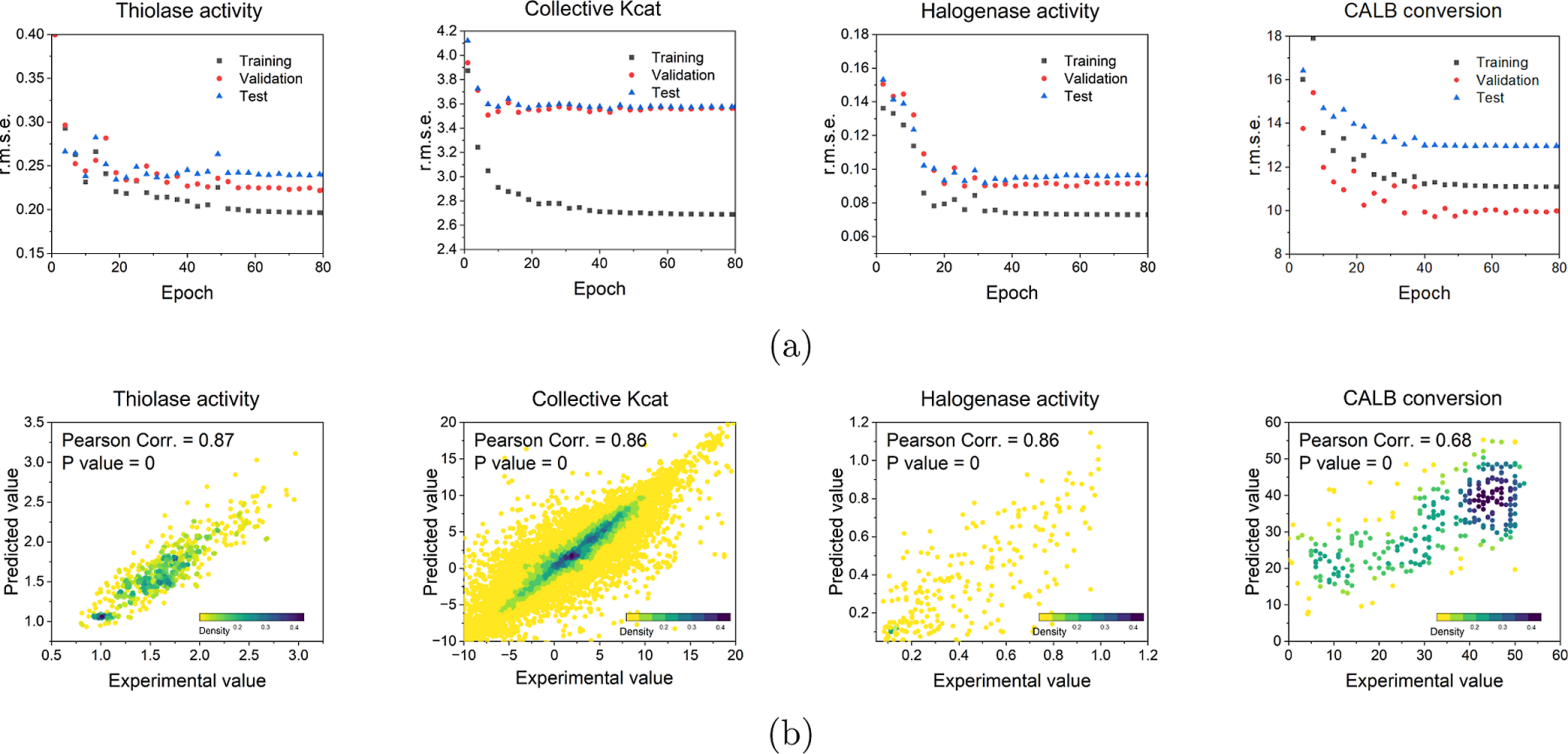
Performance of ALDELE regression models. (a) *r*.*m*.*s*.*e*. of optimal model for different data sets during the training process. (b) Prediction performance of the optimal ALDELE models. The correlation between predicted values and those present in the whole data set (training, validation, and test data sets) was evaluated. The darkness of color represents the density of data points. Student’s *t*-test was used to calculate the *P* value for Pearson’s correlation.

The predictive capacity of the optimal ALDELE models is shown in Figure 3. In the training learning curves, one epoch is one iteration of the data set passing through the NN. The *r*.*m*.*s*.*e*. values of optimal models for the test data sets, namely, thiolase activity, halogenase activity, the collective *k*_cat_, and CALB conversion data sets, are 0.22, 0.09, 3.39, and 9.99, respectively. The comparison between the prediction values and experimental values on each whole data set shows the predictive accuracy of each optimal model. The values of the Pearson’s correlation coefficient *r* are 0.87, 0.82, 0.86, and 0.68 for thiolase activity, the collective *k*_cat_, halogenase activity data sets, and the CALB conversion, respectively. The large *r*.*m*.*s*.*e*. value for the CALB data set may be attributed to scattered data distribution, unlike the thiolase and the collective *k*_cat_ data sets, in which most points were well scattered. ALDELE also demonstrates good predictivity for the Halogenase activity data set. Although the data point density is not high reflected by the poor overlapping between the test line and validation line within the narrow range, high synergy is observed at a high experimental vale range.

The structure-based machine learning methods were previously employed to predict binding affinities in drug discovery. However, these models rely on either the tertiary structures of protein–ligand complexes or pocket-ligand pairs. For instance, cutting-edge models like Pafnucy^70^ employ a 4D tensor to represent pocket-ligand structures as input features, while TopologyNet^71^ transforms 3D protein–ligand structures into one-dimensional (1D) topological fingerprints for predicting the binding affinity. Note these models are using data sets with lots of protein–ligand complex structures, such as PDBbind database^72^ (17,652 protein–ligand complexes) or CASF2016^73^ (285 protein–ligand complexes) to benchmark a protein–ligand binding affinity prediction. In contrast, for our benchmark data sets, the 3D structures of proteins are not available and have to be built by computational modeling using tools such as AlphaFold2 or Rosetta. Moreover, the above methods are dependent on the 3D structures of the protein–ligand complexes. However, to acquire reliable protein–ligand complex structures for all the enzymes in the benchmark, data sets would require large-scale molecular docking, extensive MD simulations, and sampling, and last but not least, advanced expertise for analyzing the docking and MD results, such that it is impossible to integrate these structure-based methods designed for drug discovery on the benchmark data sets for the applications in biocatalysis.

To evaluate the performance of ALDELE on CPI tasks, Table 2 summarizes a comparison of the ALDELE models and various baseline models across different data sets: thiolase activities, collective *k*_cat_, halogenase activities, and CALB conversion. It summarizes the *r*.*m*.*s*.*e*. values and coefficient of determination (*R*^2^) of test set from ALDELE models (M1 to M6) and baseline models including Random Forest (RF), Support Vector Machine (SVM), K-Nearest Neighbors (KNN) implemented with sklearn,^74^ Goldman’s ML model,^57^ Tsubaki’s,^31^ DLKcat,^56^ DeepAffinity,^33^ DeepDTA,^23^ TransformerCPI,^75^ and BACPI.^32^ The original Goldman’s KNN-based model for classification models was tailored into a regression model, which incorporated pretrained protein language features and small-molecule features for comparison with ALDELE. Tsubaki’s models were originally designed for classification tasks and have been tailored for the regression tasks here. The hyperparameters of all the models were set for a fair comparison (see Supporting Information, S5 for details of baseline methods). Compared with Tsubaki and DLKcat using single attention, M1 in part ALDELE provides a possible compared combination without attention but a pairwise module to predict interaction representation. ALDELE goes beyond the capabilities of M1 by integrating a wider range of data types. While M1 is a critical component of ALDELE, it does not, on its own, capture the entire spectrum of data. ALDELE’s ensemble approach harmonizes multiple descriptions of compounds and proteins, forming a more coherent and comprehensive predictive model. This aspect is particularly important in contrast to Tsubaki and DLKcat, which may not incorporate such a broad spectrum of data, including evolutionary information and a multifaceted attention system. The inclusion of these different models and their respective performance metrics on the same data sets allows for a direct comparison of ALDELE’s predictive performance against established methods in the field.

**Table 2:** Comparison of the ALDELE Models and the Baselines on the Data Sets of Thiolase Activities, Collective *k*_cat_, Halogenase Activities, and CALB Conversion (Regression Tasks)

data set	thiolase		collective *k*_cat_		halogenase		CALB conversion	
model	*r*.*m*.*s*.*e*.	*R*^2^	*r*.*m*.*s*.*e*.	*R*^2^	*r*.*m*.*s*.*e*.	*R*^2^	*r*.*m*.*s*.*e*.	*R*^2^
RF	0.275	0.489	4.465	0.049	0.158	0.262	16.452	–0.174
SVM	0.374	0.052	4.279	0.132	0.187	0.035	18.576	–0.473
KNN	0.282	0.521	4.462	0.130	0.157	0.079	15.669	–0.017
Goldman’s	0.294	0.477	4.463	0.141	0.148	0.293	12.972	0.271
Tsubaki’s	0.231	0.615	4.373	0.262	0.142	0.345	16.679	0.134
DLKcat	0.232	0.609	4.299	0.265	0.141	0.346	13.678	0.134
DeepAffinity	0.317	0.475	4.598	0.267	0.112	0.569	13.708	0.098
DeepDTA	0.336	0.356	4.695	0.429	0.106	0.632	13.843	0.103
BACPI	0.307	0.460	4.382	0.208	0.097	0.639	12.857	0.553
TransformerCPI	0.389	0.184	4.656	0.146	0.150	0.156	13.964	0.094
ALDELE M1	0.223	0.626	4.315	0.265	0.142	0.347	13.810	0.279
ALDELE M2	0.299	0.343	4.337	0.256	0.142	0.344	13.488	0.146
ALDELE M3	0.228	0.623	4.306	0.265	0.142	0.347	13.499	0.311
ALDELE M4	0.239	0.604	3.579	0.514	**0.092**	**0.659**	12.709	0.127
ALDELE M5	**0.222**	**0.634**	**3.396**	**0.518**	0.149	0.367	12.351	0.201
ALDELE M6	0.239	0.635			0.149	0.347	**11.108**	**0.321**

Among the models considered, the simple ML models, RF, and SVM, demonstrated suboptimal performance. Additionally, KNN was used as a nonparameric method to compare with other models. Goldman’s model represents protein sequences by pretrained featurizations ESM-1b^76^ and represented substrates by a pretrained Junction-Tree Variational Auto-Encoder (JT-VAE)^77^ with the Morgan circular fingerprints^78^ and then processed by a shallow MLP. DeepDTA and DeepAffinity are CNN-based and RNN-based methods, respectively. Tsubaki’s, DLKcat, and BACPI as well as transformerCPI are combined models of CNN and GNNs. Conversely, models employing graph-based methods demonstrate performance superior to those of CNN-based and RNN-based methods in predicting CPIs. CNN and RNN models represent compounds as strings, which may account for their worse predictive capability because the structural information on the molecules is not considered. Notably, the optimal ALDELE models achieved the lowest *r*.*m*.*s*.*e*., which indicated that it is more accurate than the baseline models for all of the data sets. The simple ML models, RF, and SVM tend to perform less effectively compared to deep learning methods, as evidenced by some negative *R*-squared values. From the comparison between Goldman’s model and the KNN model, it is evident that the introduction of pretrained protein language featurizations improves predictive performance for the mutant-based data set (CALB data set), but the improvement is not as apparent for other data sets. Incorporating pretrained features into predictive models remains a challenge that needs to be addressed. While ALDELE M1 shares the consideration of protein n-gram features and compound GNN-subgraph features with other published methods, the individual attention module employed by the Tsubaki and DLKcat models to articulate interaction representation does not exhibit performance on par with pairwise models by M1. The optimal model for thiolase activity and the collective *k*_cat_ data sets is Model5, and the optimal model for halogenase activity data sets is Model4, involving toolkits integrate a wide range of features, including compound molecular graphs, physicochemical properties, n-gram, and evolutionary information. For large-size databases, the difference of *r*.*m*.*s*.*e*. values between the optimal model and other models are minuscule. For small databases with fewer protein-compound pairs such as the CALB conversion library, the ALDELE models perform much better than the baseline models, and the optimal CPI model is ALDELE Model6. However, the large *r*.*m*.*s*.*e*. values of all evaluated models indicate the data set size is not large enough for accurate predictions.

Except for the CALB conversion data set, for which model 6 with additional structural information shows marginal improvement compared to the sequence-based models, model6 does not outperform other models for other data sets. The CALB conversion data set contains the wild-type enzyme and variants derived from the same species, and all the variant structures were built using the crystal structure of the wild-type structure as the template, whereas for other cases like thiolase and phosphatase data sets, few templates could be found with high matching scores in MSA such that the structural features directly retrieved from the predicted protein structures did not improve the models. This indicates that the structural models require careful inspection for them to represent the enzymes with a large sequence difference. Further research is needed to develop appropriate ways to incorporate structural information into a model to enhance its performance. Considering the different computing costs involved in preparing the input representations for different models, we suggest that the selection of the ALDELE toolkits could be based on comprehensive consideration of both the size of the test data set and the involved computing demand.

For enzyme screening, it would be useful to categorize enzymes into different groups based on their activities, such as those with promising activities and worthy of further engineering, as well as those with no discernible activity. To tackle these multiclass classification tasks, ALDELE was applied on the glycosyltransferase data set as a case study. We used the precision score, recall score, and F1 score to evaluate the model. The raw data were classified into zero, low, medium, and high activity classes. Unlike the previous prediction models built with binary classification formulation, in which enzymes with medium activity were considered “active”,^57^ here the activities of glycosyltransferase were classified into four different groups based on the information directly obtained from the original experiments: none, low, medium, and high activity. A precision score value of 0.86 from ALDELE M1 was obtained (Table 3), indicating the accuracy of the ALDELE multiclass classification model. The preference for M5 models in regression tasks (Table 2) and M1 models in classification tasks (Table 3) is attributed to the specific nature of the tasks and data sets employed for these tasks. M5 excels in regression tasks with complex data sets due to its more intricate toolkits, making it suitable for nuanced regression analysis. M1, however, contains simpler representations for substrates and protein sequences, is more applicable for multiclass classification tasks like glycosyltransferase data set, to effectively distinguish between discrete categories. For classification tasks, including more features may lead to overfit on the trivial information and noise in the training data, hence resulting in a poorer performance. The different performance of ALDELE models for different tasks showcases the versatility of the workflow, which ensures the choice of the most appropriate model for a specific task for the prediction of biocatalytic activities.

**Table 3:** Comparison (Precision, Recall, and F1 Scores) of the ALDELE Models on the Glycosyltransferase Data Set (a Multi-Classification Task)

model	precision score	recall score	F1 score
ALDELE M1	**0.861**	**0.886**	**0.872**
ALDELE M2	0.701	0.742	0.683
ALDELE M3	0.694	0.741	0.683
ALDELE M4	0.794	0.842	0.821
ALDELE M5	0.858	0.743	0.814

In the discussion regarding ALDELE’s performance, several key aspects of its toolkits contribute to its superior performance compared to state-of-the-art models: ALDELE’s toolkits integrate a wide range of features. The involvement of physicochemical properties (Toolkit1) and evolutionary information (Toolkit4), and structural data (Toolkit5) allows for a more nuanced understanding of the complex interplay between proteins and compounds, which is often not fully captured in simpler models. The use of a two-phase attention NN is a distinctive feature of ALDELE. This mechanism not only identifies critical areas within the protein-compound interaction landscape but also enables the model to focus on the most informative parts of the input data. By integrating multiple toolkits and leveraging the attention mechanism, ALDELE demonstrated an enhanced predictive accuracy in various tasks. This includes not only the prediction of CPIs but also the identification of enzymatic hotspots and the ability to predict biocatalyst properties. The two-phase attention mechanism in ALDELE provided the approach to capture the insights into the biological relevance of the predictions (details in the subsection). By highlighting hotspots and important interaction regions of the protein and ligand separately, ALDELE offers an interpretability layer that is often missing in other deep learning models.

In conclusion, ALDELE stands out due to its comprehensive feature integration, innovative two-phase attention mechanism, diverse toolkits, customizability, enhanced predictive accuracy, and ability to provide interpretable, biologically relevant insights. These aspects collectively contribute to its superior performance in predicting biocatalytic activities and understanding complex biomolecular interactions.

### Performance Evaluation for Fixed One-Dimension Tasks

Here, ALDELE was evaluated for fixed one-dimensional tasks in the applications of enzyme discovery or substrate discovery. For protein-only tasks, Toolkits 3 and 4 that describe protein were selected and the representation of the compound input is fixed; while for substrate-only tasks, Toolkits 1 and 2 that are based on substrates were selected and the representation of the protein input is fixed. Enzyme discovery can be applied to find new enzymes with potential activities toward a specific substrate. On the other hand, the application for substrate discovery was investigated by predicting the substrates that could be catalyzed by a certain enzyme.

**Figure 4:**
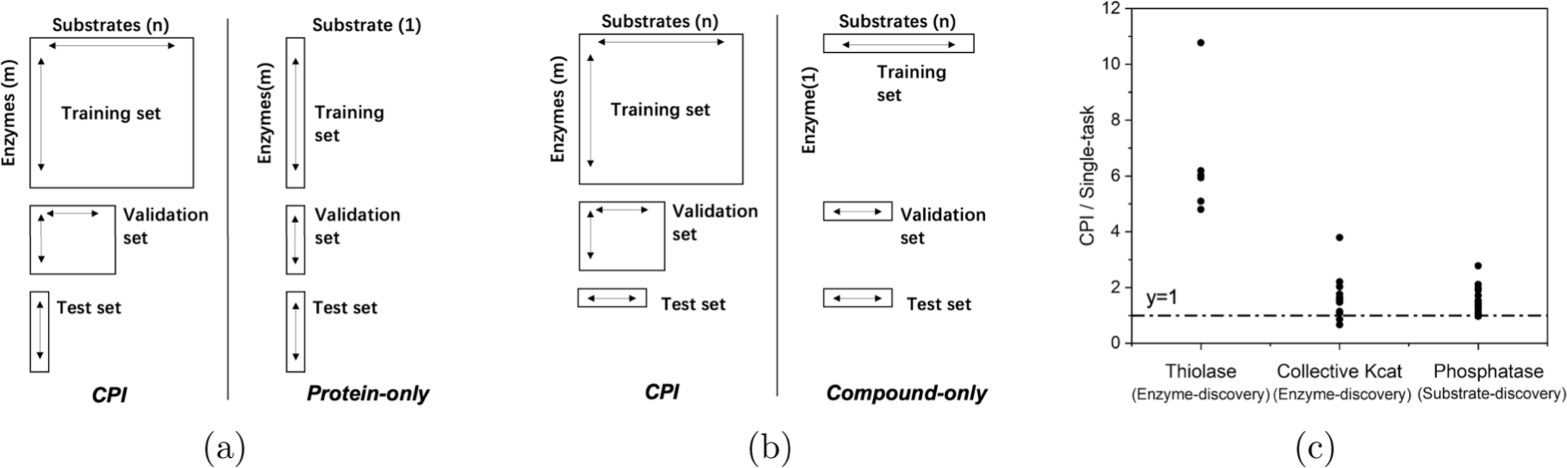
ALDELE for fixed one-dimension discovery tasks. (a) ALDELE CPI models (model 5) are compared against the fixed one-dimension models (Toolkits 3 and 4) on the data varying enzymes for a given substrate and allowing models to train on either fully expanded data or only data specific to the substrate. (b) ALDELE CPI models (model 5) are compared against the fixed one-dimension models (Toolkits 1 and 2) setting on the data varying substrates for a given enzyme and allowing models to train on either fully expanded data or only data specific to the substrate. (c) *r*.*m*.*s*.*e*. on each individual-substrate (or enzyme) task is compared between CPI models (test set results) and fixed one-dimension models (average of test set results and validation results). Points above 1 indicate for the fixed one-dimension models performing better than the CPI models in the prediction of the enzyme activities.

The comparison of data sets between the interaction task and fixed one-dimension task is shown in Figure 4a for enzyme discovery and Figure 4b for substrate discovery. Subdata sets are formed within the same data sets used for regression model testing by using those databases with sufficient data of proteins for compounds. Subdata sets for the protein-only task was generated from the thiolase activity data set that contains the sequences for one substrate and the collective *k*_cat_ data set. The fixed one-dimension substrate-only model was assessed on the phosphatase activity data set because it contains a large range of substrates (the number of substrates in each subdata set is more than 100). These subdata sets were scrutinized, and a well-balanced subdata set (a criteria set is nonzero items should be more than 30% of total data) with sufficient number of substrates was selected. The number of enzymes for the substrate-specific subdata set is more than 70.

The *r*.*m*.*s*.*e*. values for both the CPI models and the fixed one-dimension models were calculated for the selected subdata sets (Supporting Information, S6). To compare their accuracy, we calculated the ratio of the *r*.*m*.*s*.*e*. value of the CPI model to the *r*.*m*.*s*.*e*. value of the fixed one-dimension model on each subdata set (Figure 4c). Interestingly, for all three tasks (enzyme-discovery task on the thiolase activity data set or on the collective *k*_cat_ data set, and substrate-discovery task on the phosphatase activity data set), almost all ratios were on or above the horizontal line *y* = 1, indicating CPI models are inferior to the fixed one-dimension models. Remarkably, few fixed one-dimension models for the focused data sets such as the thiolase activity subdata set outperformed the CPI models for those diverse data sets, such as the collective *k*_cat_ data set, which constitutes a diverse superfamily of enzymes, and the phosphatase data set composed of a diverse range of substrates. In a previous report,^57^ the CPI models can access a larger number of enzyme–substrate interactions, and the models trained with CPIs are inferior to the models trained with the activity of enzymes on a given substrate. Our results are in agreement with the previous report, and thus, we suggest that the ALDELE models may also be expanded for predicting fixed one-dimension properties.

In our study, we analyzed CPI and one-dimensional models to understand their effectiveness in various contexts rather than asserting a universal superiority of one over the other. CPI models, which predict interactions between enzymes and substrates by considering both entities’ representations, face challenges due to the complexity of biological interactions, whereas one-dimensional models, focusing on properties of enzymes or substrates independently, show effectiveness in specific tasks. This comparison underscores the importance of both CPI and one-dimensional models in the toolbox for understanding enzyme catalytic functionality, highlighting the need for further development of CPI models to represent enzyme–substrate interactions more accurately.

### Prediction of the Hotspots for CPI

Deep learning is usually referred to as the black-box model because it is difficult to trace a prediction back to the importance of the features. Although attempts in explaining the biological relevance of the features has been made in some previous studies,^31,56^ there is no comprehensive and successful case to validate the predicted sites by experiments. ALDELE uses two-layer attention mechanisms, where the calculated attention weights can be used to identify the regions in a protein or a molecule that are important for CPI by highlighting the high-value attention weights. The regions associated with high-value attention weights are called hot spots. We conducted two groups of experiments: visualizing the binding interaction regions and the hotspot regions obtained from fixed one-dimension models.

**Figure 5:**
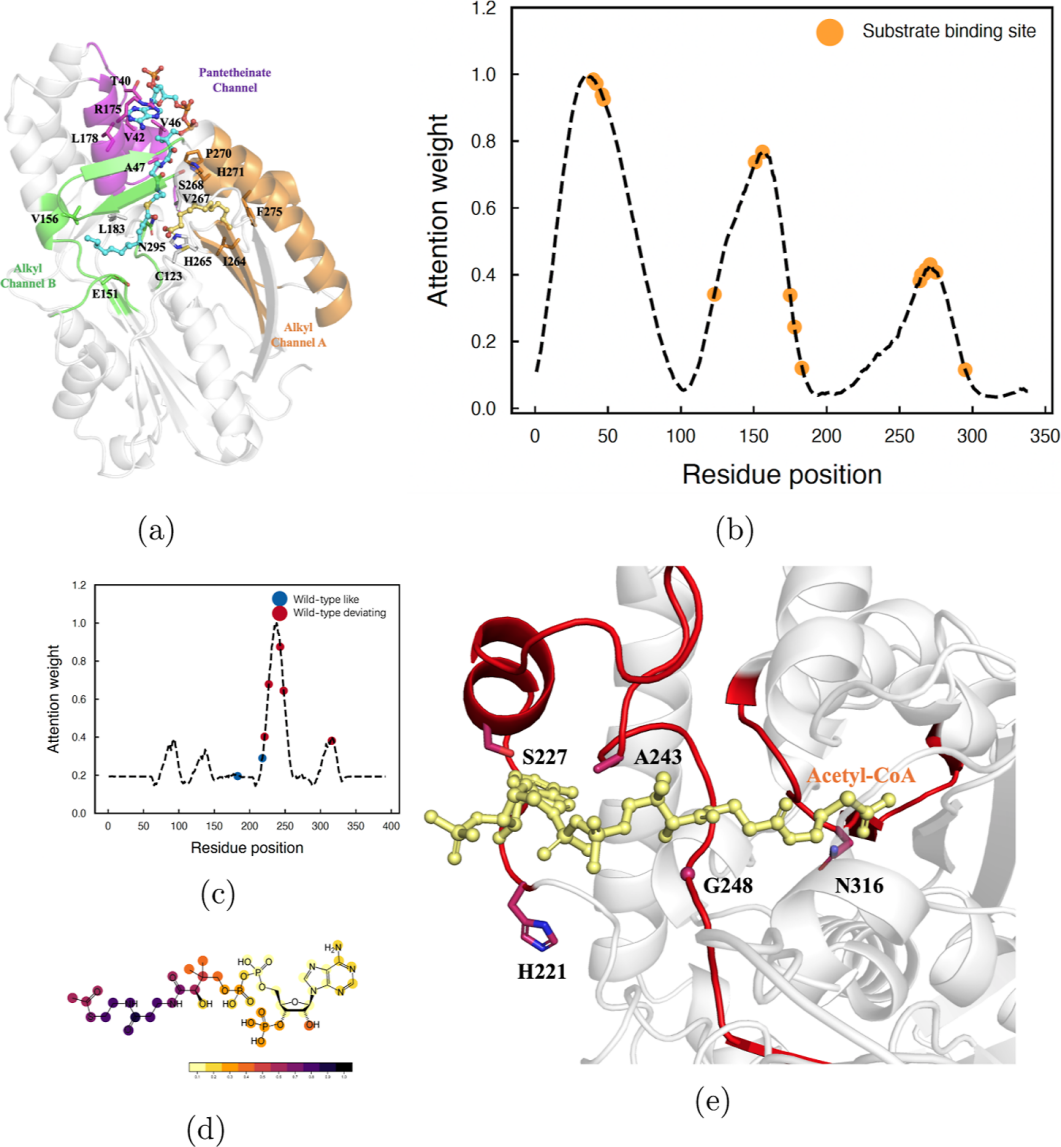
Hotspot identification with ALDELE. (a) Single monomer of OleA is shown in a gray cartoon with a pantetheinate channel (shown in purple) and two alkyl channels (shown in orange and green, respectively). The interactions of the thiolase enzyme with its substrate Acetyl-CoA are obtained from the complex crystal structure (PDB ID: 3FK5). (b) Attention weights calculated for the test thiolase enzyme. The reported interaction binding sites are labeled in orange: T40, V42, V46, A47, V91, S82, A122, C123, E151, V156, R175, R178, A179, L183, L223, I264, H265, V267, S268, P270, H271, F275, N295, G297, L323, I325, and G326. (c) Attention weights calculated for the thiolase enzyme in the complex with the substrate Acetyl-CoA (PDB ID: 1M3Z). The experimentally mutated residues of the variants are marked on the curve: mutations retaining activities compared to the wild-type enzyme (wild-type like) are shown in blue, and mutations reducing or increasing activities compared to the wild-type enzyme (wild-type deviating) are shown in red. (d) Hotspots of Acetyl-CoA with the darkness representing the attention weights of the substructures. (e) Interactions between the thiolase enzyme (PDB ID: 1M3Z) with the substrate Acetyl-CoA. The regions in proteins, which have high weight values higher than 0.5 are highlighted in red. The high-weight regions match well with the substrate binding region as shown.

ALDELE can capture the hotspot regions for test protein sequences with both high and low similarity compared with training data. The hotspot regions with high weights can be visualized by being mapped onto a known 3D protein structure. The first case study was conducted for predicting the interaction sites of thiolase with its substrate Acetyl-CoA using ALDELE. The enzyme is highly homologous (88% similarity) compared to 3-oxoacyl-(acyl carrier protein) synthase III, FabH, for which the complex crystal structure has been reported (PDB ID: 3FK5). The interaction sites reported in the experimental paper^79^ were labeled in the complex structure (Figure 5a) and the attention weight profile (Figure 5b) was obtained from ALDELE CPI model5. Most of the interaction sites sitting at the peaks have high attention weights. The attention map contained local structure information, and the highlighted regions are associated with the distribution of atom importance. This case indicates the power of ALDELE in predicting the hot-spot regions of the protein-compound interactions.

Another example is predicting the activity of a biosynthetic thiolase enzyme (PDB code: 1M3Z), which has low sequence similarity compared to any of the enzymes in the training set (similarity less than 30%). The attention weights of the test enzyme obtained from ALDELE CPI model5 are highlighted in Figure 5c. The mutation data of the enzyme–substrate pair were labeled according to the previously reported experiments,^80,81^ which are classified into the retaining and deactivating/activating mutations compared to the activity of the wild-type sequence. The interactions between the test enzyme with Acetyl-CoA are shown in Figure 5e. It shows that the regions with high attention weights correspond to the substrate binding pocket, and mutations on these sites lead to reduced enzyme activities. Most of the deactivating mutations are located at the residue positions with high attention weights, indicating the potential effect of mutation at these positions on the enzyme’s activity. On the other hand, the activity-retaining mutations are located at the regions of relatively low attention weights with values less than 0.4.

ALDELE is also able to capture the attention weights of the substrate with sequential SMILES representations of molecules. Figure 5d illustrates the compound Acetyl-CoA structure with atoms labeled to reflect the attention weights. The deepest color associates to the sixth and seventh atom subgroups (no. 4–9 atoms in SMILES string) corresponding to the atoms involved in CPI. Thus, the ALDELE model based on GNNs with compounds represented by graphs is able to predict the substructures of the substrates that can be catalyzed by a given enzyme.

### Prediction of Hotspots for Enzyme Discovery

**Figure 6:**
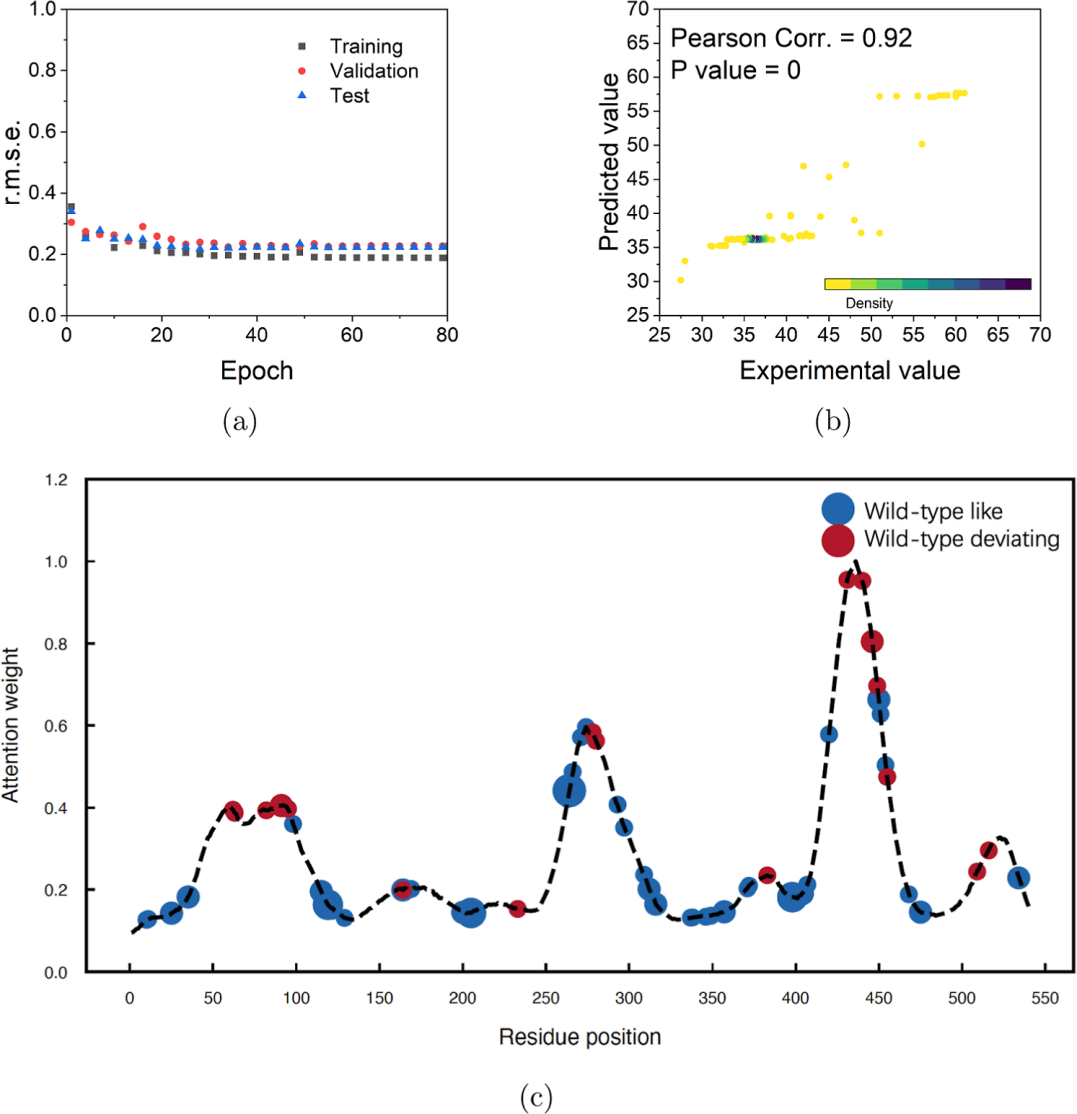
Performance of ALDELE on the BVMO thermostability data set (a) *r*.*m*.*s*.*e*. results of enzyme discovery model during the training process. (b) Performance of the model. The correlation between predicted values and those present in the whole data set (training, validation, and test data sets) was evaluated. The brightness of color represents the density of data points. Student’s *t*-test was used to calculate the *P* value for Pearson’s correlation. (c) Attention weights of the wild-type RhCHMO. The mutated residues in each of the single-mutation variants were marked on the curve: mutation retaining thermostability (wild-type like) are shown in blue, and mutations with increasing or decreasing melting temperatures (wild-type deviating) are shown in red.

The prediction performance on fixed one-dimension enzyme-discovery task was evaluated using the thermostability of the BVMO data set collected from previous experimental reports.^51−55^ The *r*.*m*.*s*.*e*. gradually decreased with increasing epoch, indicating good convergence of the training (Figure 6a). An *r*.*m*.*s*.*e*. of 2.17 was obtained for the final deep learning model trained which was used for the melting temperature prediction of the test data set. The predictive accuracy was reflected by the small deviation between the experimental and the predicted values on the entire data set (Figure 6b).

The attention weights from the deep learning model were calculated, and the AA residue positions that would have a notable effect on enzyme activity were identified, as denoted by the wild-type different residue positions with high attention weights (Figure 6c). Thus, the attention weights can be used to identify the AA positions at which mutations would have a potential effect when enzymatic properties are independent of substrates such as in the case of the thermostability prediction.

Also, ALDELE can capture attention weights to the substructure of the substrates (subgraph) and identify the atom positions that have a notable effect on their interactions with a given enzyme. To demonstrate this, the activity data set of a putative phosphatase enzyme from *E. coli* toward virous substrate molecules was used (Uniport ID E3PDZ7). We found that the weight matrix for a few randomly selected substrates from the training set displays a similar pattern.

**Figure 7:**
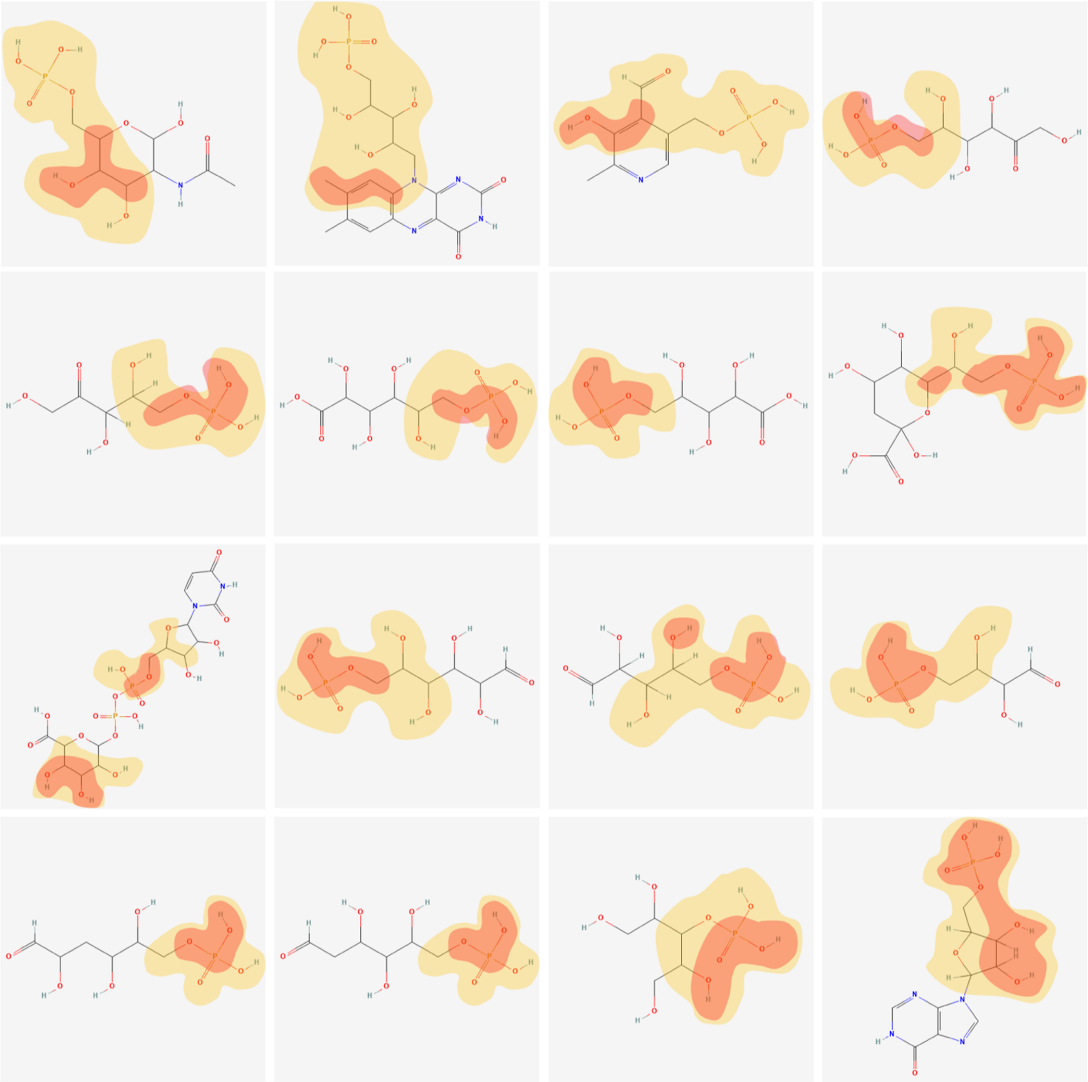
Atom importance of substrates revealed by ALDELE on phosphatase activity data set. The red area indicates the center atom of each subgroups where the model pays the highest attention, the yellow area for high attention (higher than 0.7), while the remaining gray areas with no color indicate the subgroups where the model pay less attention.

The heat map of substrate atoms is populated into three subgroups based on the attention weights (Figure 7). These clusters denote that the atom subgroups with high attention weights are located at the phosphate groups, and the second and third carbon atoms are linked to the phosphate group and the carbocyclic ring, which are the main functional groups of the substrates. The distribution demonstrates that the ALDELE can capture the local and global structure features of substrates simultaneously.

In order to evaluate the ability of ALDELE to find the patterns of substrates, we designed a special test set for the above phosphatase case. Phosphate groups are absent in the test set of substrates in contrast to the training library of substrates that have functional phosphate groups. Table 4 shows that the predicted values for the test set of the substrate without phosphate groups are much smaller than those for the substrates with phosphate groups, which aligns with our expectation (details in Supporting Information, S7).

**Table 4:** Predicted Values of a Prepared Test Set and Experimental Results of Training Set from Phosphatase Activity Data set with Different Substrates Labeled by SMILES and the Same Putative Phosphatase Enzyme from *E. coli* (Uniport ID E3PDZ7)^a^

substrates without phosphate groups (test set)	activity (%)	substrates with phosphate groups (training set)	activity (%)
CC(=O)NC1C(O)OC(CO)C(O)C1O	–0.41	CC(=O)NC1C(O)OC(COP(=O)(O)O)C(O)C1O	28.95
Cc1ncc(CO)c(C=O)c1O	8.48	Cc1ncc(COP(=O)(O)O)c(C=O)c1O	20.15
O=C(CO)C(O)C(O)C(O)CO	5.49	O=C(CO)C(O)C(O)C(O)COP(=O)(O)O	66.45
O=C(CO)C(O)C(O)CO	5.56	O=C(CO)C(O)C(O)COP(=O)(O)O	48.85
O=C(O)C(=O)CC(O)C(O)CO	–0.03	O=C(O)C(=O)CC(O)C(O)COP(=O)(O)O	42.15
O=C(O)C(O)C(O)C(O)C(O)CO	8.43	O=C(O)C(O)C(O)C(O)C(O)COP(=O)(O)O	61.85
O=C(O)C(O)C(O)C(O)CO	9.81	O=C(O)C(O)C(O)C(O)COP(=O)(O)O	59.30
O=C(O)C(O)CC(O)C(O)CO	7.63	O=C(O)C(O)CC(O)C(O)COP(=O)(O)O	57.80
O=C(O)CC(O)C(O)CO	1.14	O=C(O)CC(O)C(O)COP(=O)(O)O	54.95
O=C1OC(C(O)CO)C(O)C1(O)CO	2.92	O=C1OC(C(O)CO)C(O)C1(O)COP(=O)(O)O	49.45
O=CC(=O)C(O)C(O)C(O)CO	0.31	O=CC(=O)C(O)C(O)C(O)COP(=O)(O)O	35.20
O=CC(O)C(O)C(O)C(O)CO	10.23	O=CC(O)C(O)C(O)C(O)COP(=O)(O)O	62.30
O=CC(O)C(O)C(O)CO	12.26	O=CC(O)C(O)C(O)COP(=O)(O)O	60.60
O=CC(O)C(O)CO	0.68	O=CC(O)C(O)COP(=O)(O)O	32.25
O=CC(O)CC(O)C(O)CO	5.70	O=CC(O)CC(O)C(O)COP(=O)(O)O	52.45
O=CCC(O)C(O)C(O)CO	8.57	O=CCC(O)C(O)C(O)COP(=O)(O)O	60.95
O=CCC(O)C(O)CO	7.65	O=CCC(O)C(O)COP(=O)(O)O	44.80
OOC(C(O)CO)C(O)C(O)CO	12.99	O=P(O)(O)OC(C(O)CO)C(O)C(O)CO	31.65
OOC(CO)C(O)C(O)C(O)CO	11.05	O=P(O)(O)OC(CO)C(O)C(O)C(O)CO	46.85
OOC(CO)C(O)C(O)CO	13.78	O=P(O)(O)OC(CO)C(O)C(O)CO	30.30
OOC1OC(CO)C(O)C(O)C1O	7.93	O=P(O)(O)OC1OC(CO)C(O)C(O)C1O	41.95
OOCC(O)C(O)C(O)C(O)CO	15.31	O=P(O)(O)OCC(O)C(O)C(O)C(O)CO	63.45
OOCC(O)C(O)C(O)CCO	10.10	O=P(O)(O)OCC(O)C(O)C(O)CCO	51.90
OOCC(O)C(O)C(O)CO	20.11	O=P(O)(O)OCC(O)C(O)C(O)CO	74.40
OOCC(O)C(O)CCO	10.46	O=P(O)(O)OCC(O)C(O)CCO	55.45
OOCC(O)C(O)CO	13.11	O=P(O)(O)OCC(O)C(O)CO	64.00
OOCC(O)CO	14.15	O=P(O)(O)OCC(O)CO	51.10
OOCC1OC(O)(CO)C(O)C1O	9.66	O=P(O)(O)OCC1OC(O)(CO)C(O)C1O	43.70

a Compared to the trained substrates, the substrates in the decoy set lacks phosphate groups.

Thus, ALDELE can be employed to identify the interaction points from enzymes and their substrates for both CPI tasks and fixed one-dimension tasks.

## Conclusions

In this paper, we report a deep-learning-based multiple-toolkit framework to predict the biocatalyst properties of enzymes for specific reactions. We combined four different toolkits with four sets of features to describe enzymes and substrates, respectively. These features use either the entire properties of the compounds or proteins or the topological properties at the atomic level for compounds or AA level for proteins.

We evaluated the performance of ALDELE on various data sets with diverse sizes of pairs and demonstrated that ALDELE can be used for several applications in the development of biocatalysts. For predicting CPI tasks, ALDELE models show superior performance over the baseline state-of-the-art deep learning models on the data sets investigated. ALDELE employs the two-phase attention NN, whereby the calculated attention weights reveal the critical input regions responsible for the predicted properties, and hence can be used to identify the hot spots. For fixed one-dimensional tasks (enzyme discovery or substrate discovery), the results indicate that the models trained with both enzyme and substrate inputs are inferior to the models trained with enzymes on a given substrate. The hotspots (the atoms of compounds or the residues of proteins) with a substantial effect on CPI can also be captured. Thus, we suggest that ALDELE can also be applied for predictions for fixed one-dimension tasks and validate the applicability of ALDELE on single input tasks by evaluating the method on additional test data sets, including the phosphatase activity data set and the BVMO thermostability data set with a range of respective substrates. All of these results suggested three key points about the ALDELE method: ALDELE, utilizing deep learning representations for both compounds and proteins, is effective in predicting CPIs. This implies that the method is capable of understanding and modeling the complex interplay between the functional aspects of molecules and biomolecules; Beyond predicting interactions, ALDELE can also predict specific properties of either enzymes or substrates independently. This means that the model can focus on one type of biological entity (enzyme or substrate) and predict its properties without the need to consider the other entity. This is particularly useful in scenarios where only one side of the interaction is of interest or where information about one side is lacking; ALDELE methods with multitoolkits offers more versatility and robustness compared to traditional machine learning methods. This likely refers to ALDELE’s ability to handle a wider variety of data types (versatility).

Overall, the ALDELE method performs better for biocatalytic tasks than other competitive methods. The prediction results from ALDELE can be used to direct future enzyme engineering. Collectively, this work offers a useful end-to-end toolbox for effectively predicting CPIs with customized toolkits that users can select according to the nature of the data sets and their prediction needs, thus enabling various downstream applications such as screening functional proteins, substrate prediction, and streamlining enzymes for the biosynthesis of industrially valuable products.

## Data Availability

Source code, original data and instructions are available at: https://github.com/Xiangwen-Wang/ALDELE.

## Supporting Information Available

Downstream task details, architectural details of ALDELE models with different combination of toolkits, methods, hyperparameters of training process, machine learning methods for comparison, fixed one-dimension task models, and substrate discovery task on the phosphatase activity data set (PDF)

## Notes

ALDELE for Predicting the Biocatalytic Activities of Enzymes.
