# Supplementary material for: ALDELE: All-Purpose Deep Learning Toolkits for Predicting the Biocatalytic Activities of Enzymes
Source: J Chem Inf Model. 2024 Apr 4;64(8):3123–39. doi: 10.1021/acs.jcim.4c00058 (PMC11040732; doi:10.1021/acs.jcim.4c00058)
Supplement: Supplementary file 1 — ci4c00058_si_001.pdf [file ci4c00058_si_001.pdf]

Supplementary Information

Title: **ALDELE: All-purpose Deep Learning Toolkits for Predicting the Biocatalytic Activities of Enzymes**

Xiangwen Wang [1, 2], Derek Quinn [2], Thomas S. Moody [2,3], Meilan Huang*[1]

[1] School of Chemistry and Chemical Engineering, Queen's University Belfast, BT9 5AG, Northern Ireland, U.K.

[2] Department of Biocatalysis and Isotope Chemistry, Almac Sciences, Craigavon, BT63 5QD, Northern Ireland, U.K.

[3] Arran Chemical Company Limited, Unit 1 Monksland Industrial Estate, Athlone, Co. Roscommon, N37 DN24, Ireland

S1: Downstream task details

We used seven different datasets for CPI prediction, enzyme discovery and substrate discovery tasks. The raw dataset can be found in github.

We used four biocatalytic datasets (CALB conversion, thiolase activity, halogenase activity, and collective k_{cat} datasets) for regression tasks and one dataset (Glycosyltransferase activity dataset) for classification task to evaluate ALDELE.

CALB dataset. The conversion data on the substrates by Candida antraratica lipase B (CALB) and mutations were collected from previous experimental reports. SMILE strings of substrates are extracted from ZINC 15 database.

Thiolase dataset. The data was used and extracted as prepared by Robinson et al. for OleA enzymes in the thiolase superfamily. The activities in the original paper were obtained by calculating slopes for all overlapping 15 min intervals over the course of the first 45 min of each reaction. The log transformation was used on the maximum slope to convert the activity values in the dataset.

Halogenase dataset. The origin data was prepared by Fisher et al. by high throughput LC-MS based screening. It contains the activity of proteins against substrates on both the chlorination and bromination labels. We opted the proteins that have positive value for at least one of the substrates. The data with the bromination labels were employed because they display a more balanced percentage of active conversions.

Collective kcat dataset. The original data was generated by Li et al. with several round cleaning process. It contains the substrate name, organism information, EC number, protein identifier (UniProt ID), enzyme type and kcat values. We simplified the dataset to only keep the substrate SMILEs and protein sequences and preprocessed the dataset using the log2 transformation.

Glycosyltransferase dataset. Glycosyltransferase acceptors and donors were originally measured and classified by Yang et al. We labelled the original experimental data in 4

different classes, i.e. no, low, intermediate and high degree of activity and applied them to multi-class classification model.

Phosphatase dataset. The data was originally used by Huang et al. where the activities of 218 enzymes against 165 substrates were reported. However, many enzymes in the dataset showed no phosphatase activity to all the substrates. We selected a smaller set of 54 enzymes that displayed certain extend of activities toward the substrates. While building sub datasets for the substrate-discovery task, we further narrowed the enzyme number to 22 to ensure balanced sub datasets with R2 between 0 and 1 (a criteria set is non-zero items should be more than 30% of total data).

BVMO dataset. The BVMO thermostability dataset was built by collecting the melting temperature of wild-type and mutated enzymes in the BVMO family. This dataset only contains enzyme properties and doesn't concern substrates. It was used for enzyme-discovery task.

S2: The architectural details of ALDELE models with different combination of toolkits

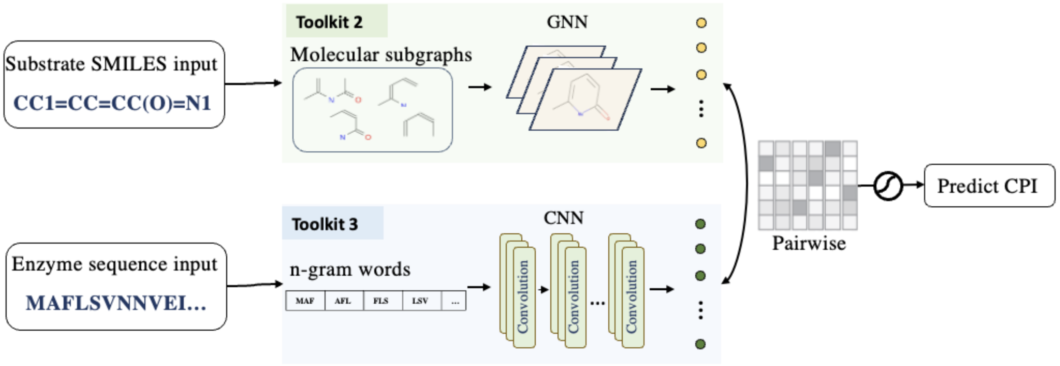

Fig. S2-1: CPI-Model 1 based on toolkits 2 and 3.

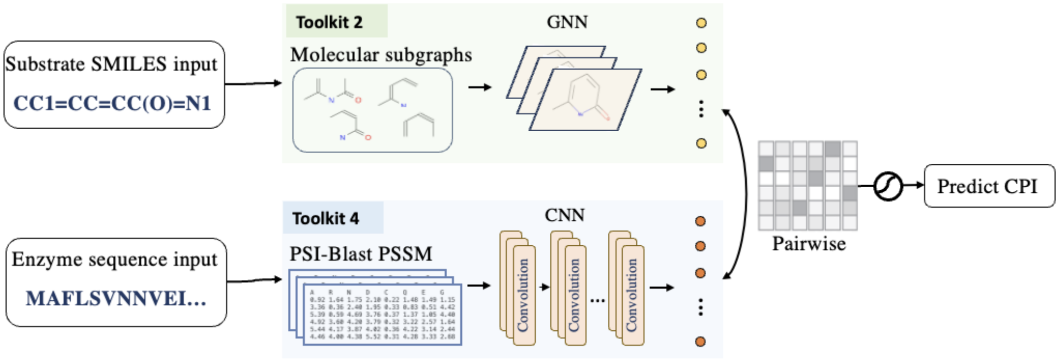

Fig. S2-2: CPI-Model 2 based on toolkits 2 and 4.

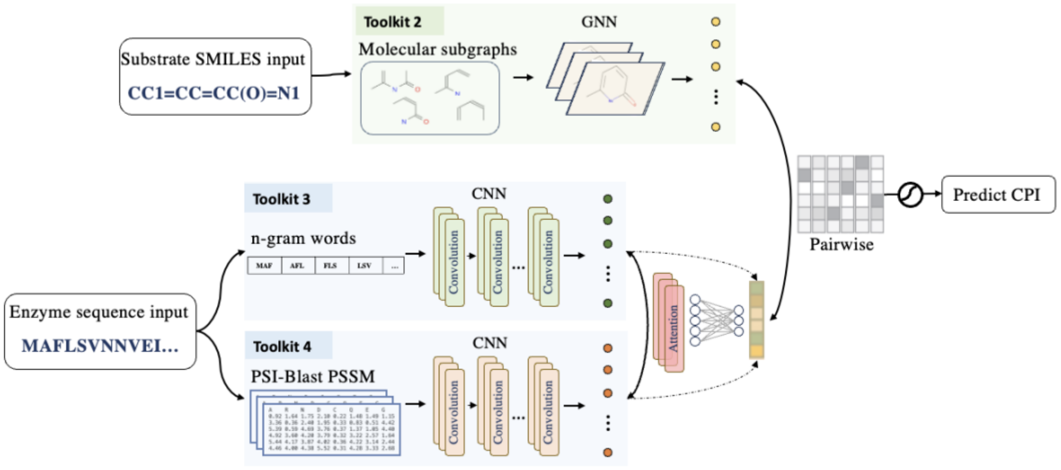

Fig. S2-3: CPI-Model 3 based on toolkits 2, 3 and 4.

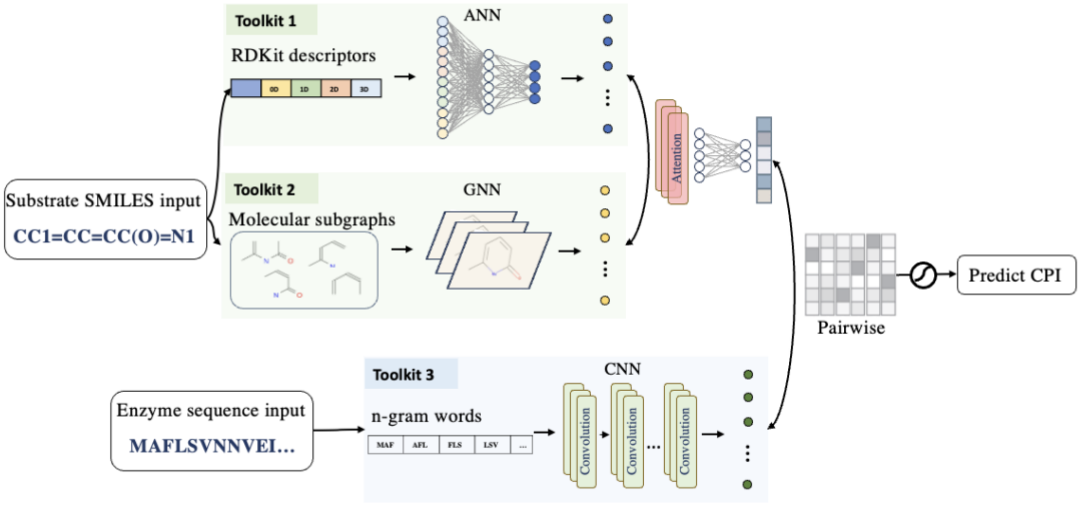

Fig. S2-4: CPI-Model 4 based on toolkits 1, 2 and 3.

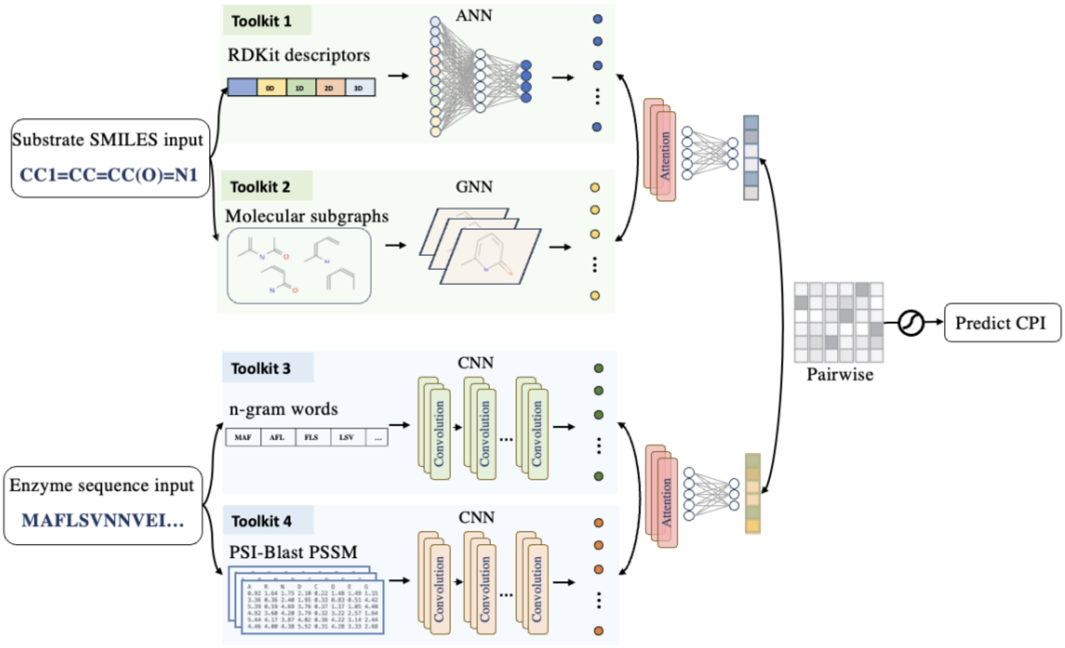

Fig. S2-5: CPI-Model 5 based on toolkits 1, 2, 3 and 4.

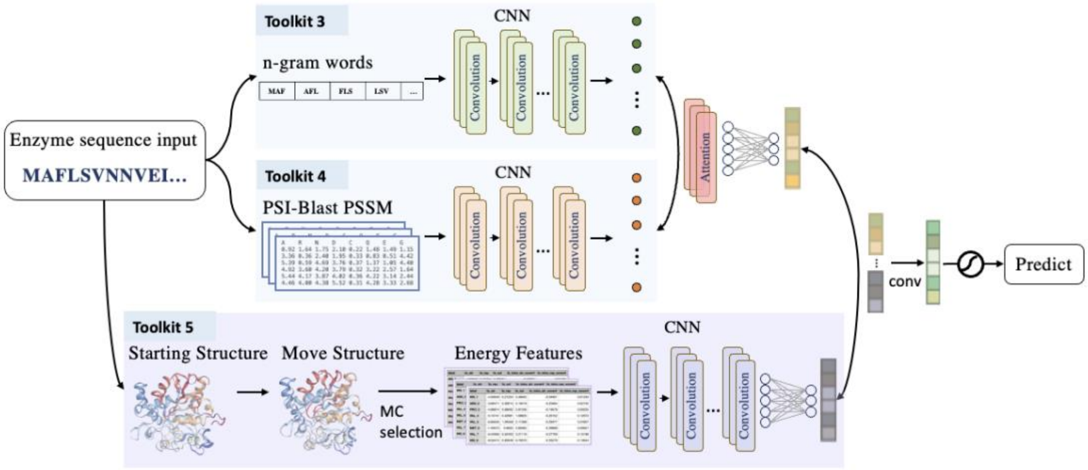

Fig. S2-6: Fix one-dimension task enzyme discovery model based on toolkits 3 and 4 or toolkits 3, 4 and 5.

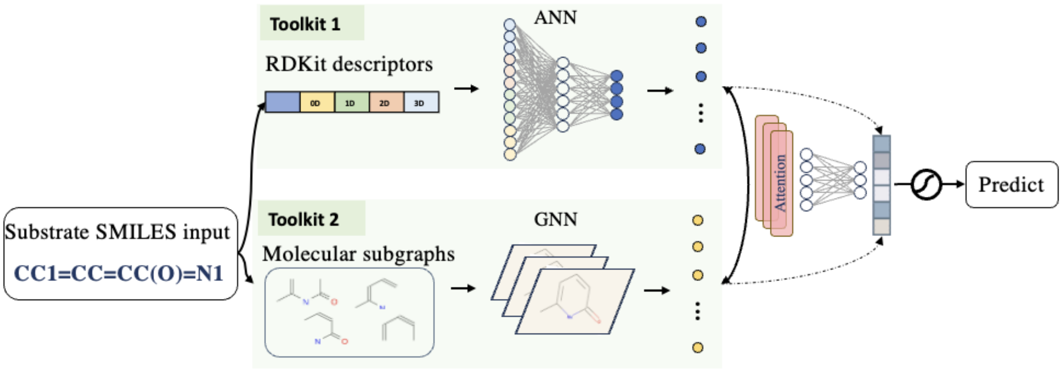

Fig. S2-7: Fix one-dimension task (substrate discovery model) based on toolkits 1 and 2.

S3: Supplementary Methods

Table S3-1: RDKit ligand descriptors computed for NN representation of substrates.

BalabanJ, BertzCT, Chi0, Chi0n, Chi0v, Chi1, Chi1n, Chi1v, Chi2n, Chi2v, Chi3n, Chi3v, Chi4n, Chi4v, EState_VSA1, EState_VSA10, EState_VSA11, EState_VSA2, EState_VSA3, EState_VSA4, EState_VSA5, EState_VSA6, EState_VSA7, EState_VSA8, EState_VSA9, ExactMolWt, FpDensityMorgan1, FpDensityMorgan2, FpDensityMorgan3, FractionCSP3, HallKierAlpha, HeavyAtomCount, HeavyAtomMolWt, Kappa1, Kappa2, Kappa3, LabuteASA, MaxAbsEStateIndex, MaxEStateIndex, MinAbsEStateIndex, MinEStateIndex, MolLogP, MolMR, MolWt, NHOHCount, NOCount, NumAliphaticCarbocycles, NumAliphaticHeterocycles, NumAliphaticRings, NumAromaticCarbocycles, NumAromaticHeterocycles, NumAromaticRings, NumHAcceptors, NumHDonors, NumHeteroatoms, NumRotatableBonds, NumSaturatedCarbocycles, NumSaturatedHeterocycles, NumSaturatedRings, NumValenceElectrons, PEOE_VSA14, RingCount, SMR_VSA1, SMR_VSA10, SMR_VSA2, SMR_VSA3, SMR_VSA4, SMR_VSA5, SMR_VSA6, SMR_VSA7, SMR_VSA9, SlogP_VSA1, SlogP_VSA10, SlogP_VSA11, SlogP_VSA12, SlogP_VSA2, SlogP_VSA3, SlogP_VSA4, SlogP_VSA5, SlogP_VSA6, SlogP_VSA7, SlogP_VSA8, TPSA, VSA_EState1, VSA_EState10, VSA_EState2, VSA_EState3, VSA_EState4, VSA_EState5, VSA_EState6, VSA_EState7, VSA_EState8, VSA_EState9, fr_Al_COO, fr_Al_OH, fr_Al_OH_noTert, fr_ArN, fr_Ar_N, fr_Ar_NH, fr_Ar_OH, fr_COO, fr_COO2, fr_C_O, fr_C_O_noCOO, fr_C_S, fr_HOCCN, fr_Imine, fr_NH0, fr_NH1, fr_NH2, fr_N_O, fr_Ndealkylation1, fr_Ndealkylation2, fr_Nhpyrrole, fr_SH, fr_aldehyde, fr_alkyl_carbamate, fr_alkyl_halide, fr_allylic_oxid, fr_amide, fr_amidine, fr_aniline, fr_aryl_methyl, fr_azo, fr_barbitur, fr_benzene, fr_bicyclic, fr_dihydropyridine, fr_epoxide, fr_ester, fr_ether, fr_furan, fr_guanido, fr_halogen, fr_hdrzine, fr_hdrzone, fr_imidazole, fr_imide, fr_isocyan, fr_isothiocyan, fr_ketone, fr_ketone_Topliss, fr_lactam, fr_lactone, fr_methoxy, fr_morpholine, fr_nitrile, fr_nitro, fr_nitro_arom, fr_nitroso, fr_oxazole, fr_oxime, fr_para_hydroxylation, fr_phenol, fr_phenol_noOrthoHbond, fr_piperdine, fr_piperzine, fr_priamide, fr_pyridine, fr_quatN, fr_sulfide, fr_sulfonamd, fr_sulfone, fr_term_acetylene, fr_tetrazole, fr_thiazole, fr_thiocyan, fr_thiophene, fr_urea, qed

Table S3-2: The features from Rosetta Score Function by amino acid position

feature	Definition
fa_atr	Lennard-Jones attractive between atoms in different residues.
fa_rep	Lennard-Jones repulsive between atoms in different residues.
fa_sol	Lazaridis-Karplus solvation energy.
fa_intra_rep	Lennard-Jones repulsive between atoms in the same residue.
lk_ball	Anisotropic contribution to the solvation.
lk_ball_bridge	Bonus to solvation coming from bridging waters, measured by overlap of the "balls" from two interacting polar atoms.
lk_ball_bridge_uncpl	Same as lk_ball_bridge, but the value is uncoupled with dGfree (i.e. constant bonus, whereas lk_ball_bridge is proportional to dGfree values).
fa_elec	Coulombic electrostatic potential with a distance-dependent dielectric.
fa_intra_elec	Intra-residue Coulombic interaction, counted for the atom-pairs beyond torsion-relationship.
hbond_sr_bb	Backbone-backbone hbonds close in primary sequence.
hbond_lr_bb	Backbone-backbone hbonds distant in primary sequence.
hbond_bb_sc	Sidechain-backbone hydrogen bond energy.
hbond_sc	Sidechain-sidechain hydrogen bond energy.
dslf_fa13	Disulfide geometry potential. Supports D- and L-cysteine disulfides, plus homocysteine disulfides or disulfides involving beta-3-cysteine.
omega	Omega dihedral in the backbone.
fa_dun	Internal energy of sidechain rotamers
p_aa_pp	Probability of amino acid at Φ/Ψ.
hxl_tors	Sidechain hydroxyl group torsion preference for Ser/Thr/Tyr, supersedes yhh_planarity (that covers L- and D-Tyr only).
ref	Reference energy for each amino acid. Balances internal energy of amino acid terms. Plays role in design.
rama_prepro	ramachandran preference

S4: Hyperparameters of training process

ALDELE: The selection of hyperparameters on deep learning performance was performed on the Thiolase activity dataset and evaluated by learning curves.

Table S4-1: Hyperparameter settings for ALDELE.

Hyperparameters	Range
Numbers of layers GNN	2; 3; 4
Numbers of layers CNN	2; 3; 4
Numbers of layers NN	2; 3; 4
R radius of subgraph	0; 1; 2; 3
N-gram of sequence	1; 2; 3; 4
RDKit descriptors dimensions	5; 10; 20
Vector dimensions	5; 10; 20
Epoch	60; 80; 100
Sliding window size	21
Learning rate	0.001
Learning rate decay	0.5
Decay interval	10
Weight decay	1e-6

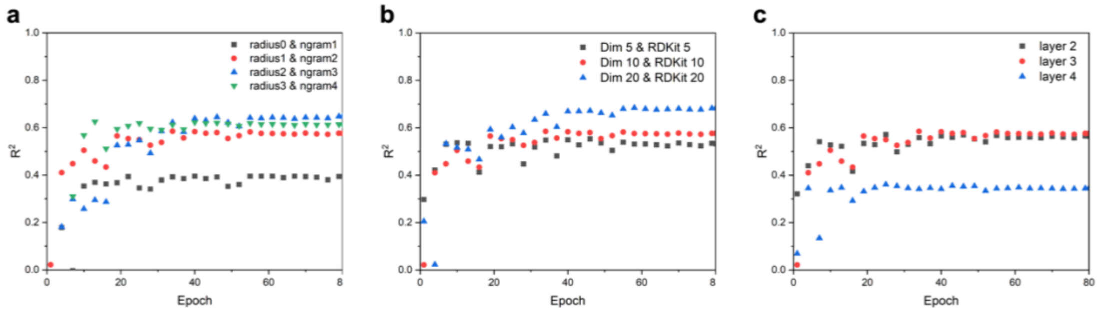

Fig. S4-1: Learning curves with various hyperparameters on the validation dataset (a) various r-radius subgraphs and n-gram amino acids, (b) various dimension vectors and top RDKit descriptors, (c) various numbers of layers in GNN, CNN and NN.

S5: Machine learning methods for comparison

The two traditional feature-based models, random forest (RF), support vector machine (SVM), and K-Nearest Neighbors (KNN) were used to compare with our approach. The training, validation and test sets in the baseline methods compared were same as those used for ALDELE methods. The most common features, molecular fingerprints from RDKit (a 208-dimensional feature vector) and protein amino acid sequence composition descriptors (an 8,567-dimensional feature vector) generated by propy }, and the PSSM features from "smooth" approach (a 420-dimensional feature vector) were all involved for a fair comparison.

The Goldman's model was tailored from original model to a KNN-based model with features, ESM-1b, a pre-trained transformer protein language featurizations (a 1280-dimensional feature vector), and Morgan fingerprint features generated by RDKit (a 1280-dimensional feature vector).

The optimized hyper-parameters of compared neural network models were taken from the original papers. Tsubaki's model hat was originally designed for classification tasks was tailored for regression tasks in this research. The hyper-parameters of these neural network models are summarized as followings:

Hyper-parameters of Tsubaki's: number of the GNN layer=3, numbers of CNN layer=3, R radius of subgraph=2, N-gram of sequence=3, vector dimensions=10, epoch=100, learning rate=0.001, learning rate decay=0.5, decay interval=10, weight decay=1e-6.

Hyper-parameters of DLKcat: numbers of layers GNN=3, numbers of layers CNN=3, R radius of subgraph=2, N-gram of sequence=3, vector dimensions=10, epoch=100, learning rate=0.001, learning rate decay=0.5, decay interval=10, weight decay=1e-6.

Hyper-parameters of DeepDTA: Numbers of layers CNN=3, Number of filters=32*1; 32*2; 32*3, Sub compound length=4, Sub sequence length=4, epoch=100,

dropout=0.1, learning rate=0.001, learning rate decay=0.5, decay interval=10, weight decay=1e-6.

Hyper-parameters of BACPI: alpha=0.1, batch size=10, vector dimensions of compound=80, dropout=0.1, gamma=0.5, number of the CNN layer=3, CNN readout=3, learning rate=0.0005, number of head=3, prot dim=80, step size=10, window=5. The default epoch of BACPI is 20, we set to 100 for a fair comparison.

Hyper-parameters of TransformerCPI: Numbers of encoder layers=3, Numbers of decoder layers=3, dimension of atom representation=64, number of attention heads=8, FFN inner hidden size=512, hidden size=64, patch size=7, learning rate=1e-4, dropout=0.2, batch size=8, weight decay=1e-4.

S6: Fixed one-dimension task models

Table. S6-1: The r.m.s.e. results using the activities dataset of Thiolase for 5 substrates-an enzyme discovery task

no.	Substrate SMILES	Dataset size	Fixed one-dimension r.m.s.e.	CPI r.m.s.e.
1	O=C(OC1=CC=C([N+]([O-])=O)C=C1)COCCOCCCC	72	0.31	1.99
2	O=C(OC1=CC=C([N+]([O-])=O)C=C1)CCCCCN=[N+]=[N-]	72	0.21	1.94
3	O=[N+](C1=CC=C(OC(CCCCC#C)=O)C=C1)[O-]	72	0.24	1.98
4	O=[N+](C1=CC=C(OC(C(C)(C)C)=O)C=C1)[O-]	72	0.46	1.45
5	CCCCCC(OC1=CC=C([N+]([O-])=O)C=C1)=O	72	0.18	1.92

Table S6-2: The r.m.s.e. results using the collective k_{cat} dataset of enzymes for various substrates – an enzyme discovery task

no.	Substrate SMILES	Dataset size	Fixed one-dimension r.m.s.e.	CPI r.m.s.e.
1	C1=NC(=C2C(=N1)N(C=N2)C3C(C(C(O3)COP(=O)(O)OP(=O)(O)OP(=O)(O)O)O)O)N	591	3.75	5.62
2	C1=CC(=C[N+](=C1)C2C(C(C(O2)COP(=O)([O-])OP(=O)(O)OCC3C(C(C(O3)N4C=NC5=C(N=CN=C54)N)O)O)O)O)C(=O)N	392	3.45	5.85
3	C1C=CN(C=C1C(=O)N)C2C(C(C(O2)COP(=O)(O)OP(=O)(O)OCC3C(C(C(O3)N4C=NC5=C(N=CN=C54)N)OP(=O)(O)O)O)O)O	301	4.01	5.90
4	C1C=CN(C=C1C(=O)N)C2C(C(C(O2)COP(=O)(O)OP(=O)(O)OCC3C(C(C(O3)N4C=NC5=C(N=CN=C54)N)O)O)O)O	273	4.12	5.41
5	C1=CC(=C[N+](=C1)C2C(C(C(O2)COP(=O)(O)OP(=O)(O)OCC3C(C(C(O3)N4C=NC5=C(N=CN=C54)N)OP(=O)(O)O)O)O)O)C(=O)N	241	4.31	6.95
6	C(CC(=O)O)C(C(=O)O)N	170	3.31	5.16
7	CC(=O)SCCNC(=O)CCNC(=O)C(C(C)(C)COP(=O)(O)OP(=O)(O)OCC1C(C(C(O1)N2C=NC3=C(N=CN=C32)N)O)OP(=O)(O)O)O	160	6.49	5.10
8	C(C1C(C(C(C(O1)O)O)O)O)O	148	2.26	5.03
9	CC(=O)C(=O)[O-]	142	3.61	4.15
10	C(CC(=O)O)C(=O)C(=O)O	121	3.87	3.55
11	C1=CC=C(C=C1)CC(C(=O)O)N	115	2.35	4.15
12	C1C(C(C(C(O1)O)O)O)O	113	3.00	5.36
13	C(C(C(=O)O)N)C(=O)O	109	3.31	6.71
14	C1C2CN(CN2C3=C(N1)N=C(NC3=O)N)C4=CC=C(C=C4)C(=O)NC(CCC(=O)O)C(=O)O	108	2.15	8.12
15	C[S+](CCC(C(=O)[O-])N)CC1C(C(C(O1)N2C=NC3=C(N=CN=C32)N)O)O	106	2.51	4.17
16	CC(C(=O)O)N	106	3.80	4.12

Table S6-3: The r.m.s.e. results using the activities of Phosphatase dataset - an enzyme discovery task

no.	Protein ID	Protein family	Dataset size	Fixed 1D task r.m.s.e.	CPI r.m.s.e.
1	P0A8Y5	Escherichia coli (strain K12)	108	0.133	0.14
2	Q97DU2	Clostridium acetobutylicum	108	0.181	0.234
3	Q97JQ5	Clostridium acetobutylicum	108	0.181	0.201
4	A6L7P8	Bacteroides vulgatus	108	0.162	0.211
5	Q9K9F5	Bacillus halodurans	108	0.201	0.241
6	Q819D3	Bacillus cereus	108	0.213	0.214
7	A6B4T0	-	108	0.148	0.178
8	Q836C7	Enterococcus faecalis	108	0.287	0.397
9	Q88LM3	Pseudomonas putida	108	0.139	0.21
10	O08430	Salmonella typhi	108	0.096	0.237
11	P68522	Bacillus subtilis	108	0.147	0.179
12	Q03SW9	Lactobacillus brevis	108	0.155	0.201
13	E3PDZ7	Escherichia coli	108	0.147	0.215
14	A5LVA8	Streptococcus pneumoniae	108	0.236	0.297
15	Q49VS1	Staphylococcus saprophyticus	108	0.125	0.21
16	C2JBT4	Vibrio cholerae	108	0.188	0.239
17	Q9RR83	Deinococcus radiodurans	108	0.158	0.201
18	Q97T51	Streptococcus pneumoniae	108	0.19	0.353
19	P21829	Escherichia coli	108	0.145	0.17
20	Q5M5S4	Streptococcus thermophilus	108	0.195	0.298
21	Q9KMS7	Vibrio cholerae serotype	108	0.188	0.28
22	P27848	Escherichia coli	108	0.205	0.247

S7: Substrate discovery task on phosphatase activity dataset

Table S7-1: 16 substrates chosen from the phosphatase activity dataset for calculating the attention weights.

No.	Substrate name	PubChem CID
1	Methyl dihydrogen phosphate	898
2	Riboflavine 5'-phosphate	710
3	Pyridoxal phosphate	1051
4	DL-Fructose-6-phosphate	603
5	Pentulose, 5-phosphate	850
6	d-Gluconic acid, 6-(dihydrogen phosphate), trisodium salt	422
7	2,3,4-Trihydroxy-5-phosphonooxypentanoic acid	3283969
8	2,4,5-Trihydroxy-6-(1-hydroxy-2-phosphonooxyethyl)oxane-2-carboxylic acid	3370098
9	UDP-a-D-Galacturonic acid	390
10	D-Glucose 6-phosphate	99058
11	5-o-Phosphonopentose	230
12	Tetrose, 4-phosphate	697
13	(2,3,5-Trihydroxy-6-oxohexyl) dihydrogen phosphate	14870
14	(2,3,4-Trihydroxy-6-oxohexyl) dihydrogen phosphate	5083977
15	1,2,4,5-Tetrahydroxypentan-3-yl dihydrogen phosphate	91134279
16	Polyinosinic acid	135402036

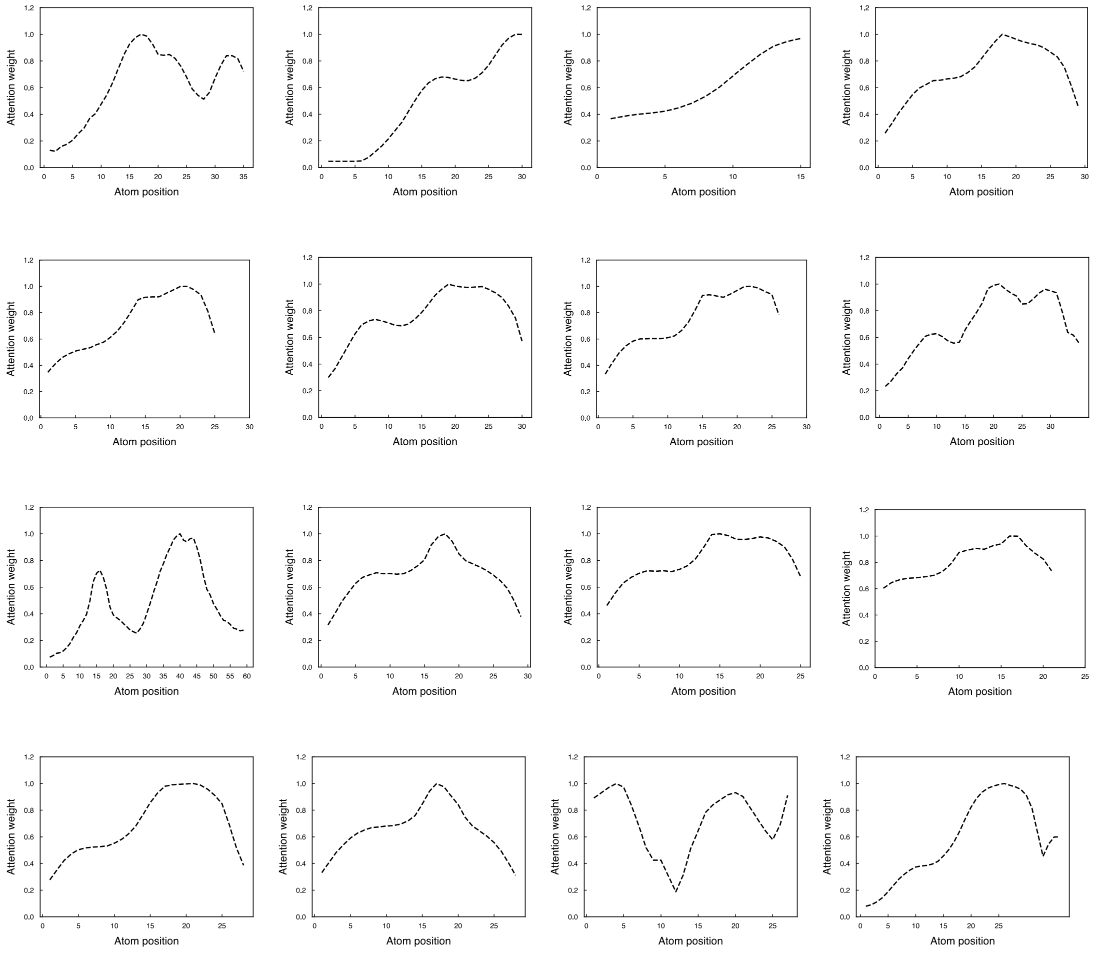

Fig. S7-1: Attention weights of the example molecule of phosphatase activity dataset.
